# Patient‐Centred Communication About AI‐Related Information in Perioperative Care: An Attention‐Controlled Randomised Trial of Anxiety, Perceived Control and Interpretive Preparedness

**DOI:** 10.1111/hex.70838

**Published:** 2026-11-13

**Authors:** Volkan Gökmen, Burcu Demir Gökmen

**Affiliations:** ^1^ Department of Nursing, Faculty of Health Sciences Ağrı İbrahim Çeçen University Ağrı Türkiye

**Keywords:** artificial intelligence, health literacy, patient and public involvement, patient–clinician communication, perceived control, perioperative care, preoperative anxiety, randomised controlled trial

## Abstract

**Background or Context:**

Patients encountering artificial intelligence (AI) in health care may be uncertain about whether AI supports or replaces clinicians, who remains accountable and how general AI‐related information applies to their own care. These are questions about how clinicians talk about AI rather than about how AI performs, and they may be particularly salient before surgery. This trial evaluated communication about AI‐related information only: no AI system, clinical algorithm, device or decision‐support tool was used, tested or applied to any participant's care.

**Objective:**

The aim of this study was to evaluate whether a brief, public‐informed, clinician‐delivered protocol of structured AI communication improves immediate interpretive preparedness before surgery compared with time‐matched attention‐control supportive communication.

**Design:**

Single‐centre, two‐arm, parallel‐group, attention‐controlled superiority randomised trial with 1:1 allocation.

**Setting and Participants:**

Adult patients scheduled for elective surgery at Ağrı Training and Research Hospital, Ağrı, Türkiye, were randomised 1:1 to structured AI communication (*n* = 60) or attention‐control supportive communication (*n* = 60).

**Intervention:**

Structured AI communication was a 5–7‐min nurse‐delivered session explaining that AI may support but does not replace health professionals, that responsibility for clinical decisions remains with the care team, that AI‐related information may be general or probabilistic, and that such information should be interpreted within the patient's own clinical context. Attention‐control supportive communication was time‐matched nurse–patient contact about routine perioperative preparation without AI‐specific content.

**Main Outcome Measures:**

The primary outcome was pre‐surgery anxiety measured using the Amsterdam Preoperative Anxiety and Information Scale (APAIS) anxiety subscale. The key secondary outcome was perceived control. AI‐related uncertainty, information clarity and trust in the clinical team were measured immediately after the session as study‐specific communication‐uptake indicators rather than as validated independent clinical endpoints.

**Results:**

All 120 randomised participants completed the pre‐surgery endpoint assessment. Pre‐surgery anxiety was lower after structured AI communication than after attention‐control supportive communication (9.28 ± 2.55 vs. 12.48 ± 3.01; baseline‐adjusted mean difference [MD], −2.56; 95% CI, −2.87 to −2.26; *p* < 0.001). Structured AI communication was also associated with higher perceived control (25.83 ± 4.04 vs. 22.62 ± 3.90; adjusted MD, 3.73; 95% CI, 3.34 to 4.12; *p* < 0.001), greater information clarity and trust, and lower AI‐related uncertainty. Clinically meaningful anxiety reduction (≥ 2 APAIS points) was more frequent after structured AI communication (48/60, 80.0%) than after attention‐control supportive communication (1/60, 1.7%). The unblinded design and the immediate self‐reported assessment may have amplified these differences through expectancy, novelty, social desirability and measurement reactivity. The responder result should therefore be interpreted as a short‐term self‐reported difference rather than evidence of durable clinical benefit.

**Discussion and Conclusions:**

In a single centre, a brief public‐informed protocol was associated with better immediate self‐reported psychological and interpretive preparedness before surgery. Structured AI communication about AI‐related uncertainty and human accountability is a plausible patient‐centred component of technology‐enabled care. These findings do not show that AI technology itself improves patient outcomes, and they do not establish durable anxiety reduction, behaviour change or clinical benefit beyond the immediate preoperative window.

**Lived Experience or Public Contribution:**

Three lived‐experience advisors reviewed patient‐facing materials during protocol refinement. After analysis, a six‐member public advisory group reviewed plain‐language findings and advised on interpretation, practitioner‐facing wording and calibrated trust, accountability and human oversight. Their feedback informed the Discussion and Practitioner Points.

**Patient and Public Contribution:**

Three lived‐experience advisors with previous sur cal experience reviewed the patient‐facing communication materials during protocol refinement. After analysis, a six‐member public advisory group reviewed a plain‐language summary of the findings and informed interpretation, practitioner‐facing wording and the framing of calibrated trust, accountability and human oversight.

**Trial Registration:**

This randomised controlled trial was prospectively registered at ClinicalTrials.gov (Identifier: NCT07393945).

## 1 Introduction

Artificial intelligence (AI) is increasingly visible in health care both as technical infrastructure for clinicians, organisations and health systems and as a topic patients encounter through public debate, digital media, online health information and clinical communication [1, 2, 3]. The patient‐facing consequences of AI are not limited to whether an algorithm is accurate or clinically effective. Patients may also need to understand whether AI supports or replaces health professionals, who remains accountable for decisions and how general AI‐related information should be interpreted in an individual clinical context.

Responsible‐AI frameworks emphasise transparency, explainability, accountability, safety, equity and human oversight, but express these principles in technical, regulatory or organisational language [4, 5]. For patients, the same principles surface as practical concerns about clinician replacement, professional responsibility, trust and personal relevance, and acceptance of AI in health care appears closely linked to whether AI is understood as supporting, rather than replacing, human clinical judgement [6, 7, 8, 9].

The perioperative setting is a clinically important context in which to examine these communication challenges. Patients preparing for surgery commonly face uncertainty about anaesthesia, operative risk, postoperative pain, recovery and complications, and preoperative anxiety is frequent and may affect patient experience, information processing and psychological preparedness [10, 11, 12, 13]. Standard preoperative education focuses on procedural preparation, fasting, anaesthesia, pain management and recovery expectations; it does not usually include structured explanation of AI‐related information, uncertainty, accountability or human oversight, even though patients increasingly encounter AI‐related claims through online sources, media narratives and technology‐enabled care.

Communication about uncertainty provides a conceptual foundation for this problem. Uncertainty in health care may arise from probability, ambiguity, complexity, limited evidence or the difficulty of applying general information to an individual case [14, 15]. Effective uncertainty communication does not remove uncertainty; it helps patients understand what is known, what remains uncertain and who is responsible for interpreting information in the context of care [16]. This distinction is particularly important for AI, because communication that simply reassures patients that AI is safe may encourage overtrust, whereas communication that emphasises uncertainty without context may increase anxiety or distrust. A patient‐centred approach should therefore support calibrated trust by clarifying that AI‐related information is limited, contextualised and interpreted within accountable, clinician‐led care.

Perceived control and digital health literacy may further shape patients' responses to AI‐related communication. In the perioperative context, perceived control refers not to control over surgical or anaesthetic decisions but to orientation, predictability, understanding and the ability to ask questions within the care process [17, 18]; patients also differ in their ability to locate, evaluate and apply digital health information, which may influence how they interpret AI‐related claims [19, 20, 21, 22]. These issues connect with broader frameworks of patient engagement, shared decision‐making and patient‐centred care [23, 24, 25, 26], and they make patient and public involvement especially important: the acceptability and clarity of explanations about AI, uncertainty and human oversight depend on how patients and members of the public interpret the language professionals use, and involvement in digital health work is often inconsistently reported or limited in depth [27, 28, 29, 30, 31].

Despite an expanding literature on AI ethics, regulation, implementation and public attitudes, interventional evidence on how to communicate with patients about AI in clinical care remains scarce. Existing studies have largely examined attitudes, acceptability, willingness to use AI, or concerns about privacy, safety and clinician replacement [6, 7, 8, 9]. Much less is known about whether a brief structured AI communication protocol can improve patient‐centred outcomes in a real clinical setting, particularly before surgery.

The objective of this attention‐controlled randomised trial was to determine whether structured AI communication, defined as brief patient‐centred clarification of AI‐related information, uncertainty, professional accountability and human oversight, improves immediate interpretive preparedness before surgery compared with time‐matched attention‐control supportive communication. The primary outcome was pre‐surgery anxiety, the key secondary outcome was perceived control, and AI‐related uncertainty, information clarity and trust in the clinical team were assessed as proximal communication‐uptake indicators. We hypothesised that structured AI communication would reduce pre‐surgery anxiety and improve patient‐centred indicators of interpretive preparedness relative to attention‐control supportive communication.

## 2 Methods

### 2.1 Study Design, Registration and Reporting

This was a single‐centre, two‐arm, parallel‐group, attention‐controlled superiority randomised trial with a 1:1 allocation ratio. The study was designed and reported in accordance with the CONSORT 2010 statement for parallel‐group randomised trials and the CONSORT extension for non‐pharmacological treatments [32, 33]; the intervention is additionally described against the TIDieR framework in Supplementary Appendix Section A [34]. The trial was prospectively registered at ClinicalTrials.gov (Identifier: NCT07393945), ethical approval was obtained from the Ağrı İbrahim Çeçen University Scientific Research Ethics Committee (Decision No. 56, approval date: 29 January 2026), and all participants provided written informed consent before enrolment. No changes to eligibility criteria, trial design, intervention content, primary outcome or main analysis plan were made after trial commencement.

The trial evaluated communication about AI‐related information as a component of perioperative preparation. It did not evaluate the technical performance, diagnostic accuracy, safety or clinical effectiveness of an AI system, device, diagnostic algorithm or decision‐support tool, and no AI system was applied to participants' care. This distinction defined the scope of the intervention, outcome interpretation and conclusions.

### 2.2 Setting, Recruitment and Participants

The trial was conducted at Ağrı Training and Research Hospital, Ağrı, Türkiye, during routine preoperative preparation. Recruitment took place between 05 February 2026 and 25 April 2026. Because the primary endpoint was assessed before surgery, follow‐up for each participant ended at the pre‐surgery endpoint assessment. No pre‐screening filter was applied to the elective operating list: every consecutive adult patient scheduled for elective surgery during the recruitment window was approached, so the 138 patients recorded as assessed for eligibility represent the complete consecutive series rather than a pre‐selected subset. Patients were eligible if they were aged 18 years or older, able to provide written informed consent and able to complete the study questionnaires during the preoperative period. Patients were excluded if they were unable to complete questionnaires, had urgent clinical circumstances preventing participation in a brief communication session, or had cognitive, communication or clinical limitations precluding informed participation; the reasons recorded for each ineligible patient are summarised in Supplementary Appendix Table D1. Baseline assessment was completed before randomisation and before delivery of either communication condition.

Several design features plausibly explain the high enrolment fraction and the complete absence of attrition, and they should be read as constraints on interpretation rather than as indicators of engagement. Participation required a single 5–7‐min conversation and three brief self‐administered questionnaires completed in the preoperative area; there was no biological sampling, no medication exposure, no additional hospital visit and no post‐discharge follow‐up; the endpoint occurred on the same day, before surgery, while participants were already waiting in the unit; and a research nurse remained available throughout. Because the final assessment preceded the operation, no participant had any interval in which to be lost to follow‐up after randomisation. Retention here therefore reflects the brevity and timing of the protocol, not sustained engagement, and says little about willingness to take part in longer or more demanding studies of AI‐related communication.

### 2.3 Public and Lived‐Experience Contribution to Intervention Development and Interpretation

Public and lived‐experience contribution was incorporated as a structured consultative component to ensure that the protocol addressed patient‐facing interpretive concerns rather than only technical or clinician‐defined priorities. The intervention was designed to translate responsible‐AI principles, including transparency, accountability, uncertainty and human oversight, into brief, understandable and non‐promotional perioperative communication. During protocol refinement, three independent lived‐experience advisors with previous surgical experience reviewed the patient‐facing materials for clarity, acceptability and patient relevance. Their feedback helped ensure that AI‐related information was described as supportive rather than substitutive of professional judgement and that the wording did not imply autonomous, definitive or patient‐specific AI decision‐making. After completion of the primary analysis, a six‐member public advisory group with previous elective surgery experience reviewed a plain‐language summary of the findings, completed a structured feedback form and participated in a facilitated discussion. This post‐analysis stage focused on interpretation, the patient‐level meaning of anxiety reduction, practitioner‐facing recommendations and the framing of calibrated trust, accountability and human oversight. Public feedback informed revisions to the Discussion and Practitioner Points, particularly by supporting the presentation of the intervention as improving interpretive preparedness rather than promoting AI or implying that AI technology itself improves outcomes. This contribution was consultative rather than full co‐production: public contributors did not participate in recruitment, randomisation, intervention delivery, outcome assessment, statistical analysis or trial governance, and they did not alter predefined outcomes or the analysis plan. The specific influence of public and lived‐experience contribution is summarised in Table 1, with full documentation in Supplementary Appendix Section B.

**Table 1:** Public and lived‐experience contribution and its influence on the study.

Study stage	Public/lived‐experience contribution	Influence on intervention, interpretation or manuscript
Identification of patient‐facing communication needs	Lived‐experience advisors confirmed that patients may be uncertain about whether AI supports or replaces clinicians, who remains accountable and whether general AI‐related information applies to their own care.	The intervention was framed as an interpretive preparedness protocol rather than as education about a specific AI system.
Protocol refinement	Three independent lived‐experience advisors with previous surgical experience reviewed the patient‐facing communication materials.	Technical and promotional wording was reduced; the protocol more clearly stated that AI may support but does not replace doctors, nurses or the clinical team.
Accountability wording	Advisors reviewed whether the communication clearly explained professional human oversight and continuing clinical responsibility.	The final script explicitly stated that clinical decisions remain the responsibility of qualified health professionals and are interpreted according to the patient's own condition, history and surgical plan.
Communication boundaries	Advisors commented on wording that could make AI appear overly certain, autonomous or personally directive.	Prohibited wording was defined, including statements implying that AI decides care, is always accurate or applies directly to the individual patient without clinical interpretation.
Post‐analysis interpretation	A six‐member public advisory group with previous elective surgery experience reviewed a plain‐language summary of the findings and discussed their meaning.	The Discussion was revised to frame the findings as immediate interpretive preparedness and calibrated trust rather than as evidence that AI technology itself improves outcomes.
Practitioner‐facing recommendations	The public advisory group reviewed the clarity and usefulness of practitioner‐facing messages.	Practitioner Points were shortened and rewritten in directive language focused on AI's supportive role, professional accountability and invitation to patient questions.
Future research priorities	Public advisors raised data privacy as a concern during discussion.	Data privacy was documented as a patient‐relevant issue for future research, but not presented as a claim of the present trial because data governance was outside its scope.

### 2.4 Sample Size and Power

The sample size was determined for the primary outcome: pre‐surgery anxiety measured using the Amsterdam Preoperative Anxiety and Information Scale anxiety subscale [35]. Detection of a 2.0‐point between‐group difference was considered clinically meaningful for this trial because such a change represents a visible shift on the APAIS anxiety scale and was judged by the study team and public contributors to be meaningful in the immediate preoperative context. Assuming a common standard deviation of 2.7, a two‐sided alpha level of 0.05 and equal allocation, a total sample size of 120 participants (60 per group) provided more than 90% statistical power to detect a 2.0‐point between‐group difference in the primary endpoint. The recruitment target was met exactly, and all randomised participants completed the primary endpoint assessment.

### 2.5 Randomisation, Allocation Concealment and Masking

After baseline assessment, participants were randomised 1:1 to structured AI communication or time‐matched attention‐control supportive communication. An independent statistical consultant, not involved in recruitment, intervention delivery, outcome assessment or clinical care, generated the randomisation sequence. Allocation was concealed using sequentially numbered, sealed, opaque envelopes opened only after eligibility confirmation and baseline questionnaire completion. Concealment was recorded as adequate for all randomised participants. Because this was a face‐to‐face behavioural communication intervention, participants and nurses could not be blinded to group assignment. To reduce performance, detection and analytical bias, both groups received time‐matched communication, delivery was standardised using scripts and checklists, outcomes were self‐administered and primary/sensitivity analyses used coded group labels before allocation interpretation. Protocol deviation logs and allocation‐concealment records were retained, and no randomisation‐ or allocation‐related protocol violations were recorded.

### 2.6 Intervention Development

The structured AI communication protocol was developed to translate core responsible‐AI principles into brief, patient‐facing, non‐technical language. It focused on five predefined components: AI as supportive rather than substitutive; professional human oversight; continuing clinical responsibility; uncertainty and generalisability of AI‐related information; and contextualisation within the patient's own clinical situation. These components addressed common patient‐facing concerns about whether AI replaces clinicians, who remains responsible, whether AI‐related information is definitive or probabilistic and whether general digital information applies directly without professional interpretation. Consultative input from lived‐experience advisors informed the final wording and communication boundaries, including simplification of technical language, avoidance of promotional framing and emphasis on professional accountability. The final protocol was framed as general clarification about AI‐related information and did not imply that an AI system was being used to make decisions about the participant's own surgery.

### 2.7 Structured AI Communication Intervention

Participants allocated to the intervention group received a trained nurse–delivered, face‐to‐face communication protocol lasting approximately 5–7 min. The session was conceptualised as an interpretive preparedness intervention to help patients understand what AI‐related information can and cannot mean, who remains responsible for clinical decisions and how general AI‐related information should be interpreted in relation to their own care. The protocol addressed five elements: AI as supportive rather than substitutive; professional responsibility for clinical decisions; the general, probabilistic or uncertain nature of AI‐related information; the need for professional contextualisation of online or AI‐supported information; and encouragement to ask questions about AI, uncertainty, accountability or perioperative care. The full script, permitted flexible wording, prohibited content and delivery checklist are provided in Supplementary Appendix Section A.

### 2.8 Attention‐Control Comparator

Participants allocated to the comparator group received standard preoperative information plus time‐matched supportive nurse–patient communication. The comparator controlled for non‐specific effects of nurse attention, interpersonal contact, communication time and the opportunity to ask general perioperative questions, while excluding AI‐specific explanatory content. It included supportive clarification about routine perioperative preparation but did not discuss AI, AI‐related uncertainty, professional oversight of AI‐supported systems or contextualisation of general AI‐related information. The comparator script and attention‐control wording boundaries are provided in Supplementary Appendix Section A.

### 2.9 Training, Standardisation and Process Monitoring

Nurses delivering the communication received structured orientation covering protocol components, patient‐centred language, permitted and prohibited wording, time equivalence between groups and procedures for responding to questions without introducing non‐protocol content. Flexible wording was permitted within predefined boundaries, provided that the core meaning of each component was preserved. Standardisation was supported through written scripts, checklist‐guided delivery and process documentation; for each session, providers recorded session duration, nurse identifier, item‐by‐item checklist completion and the interval between the immediate post‐communication assessment and the pre‐surgery endpoint.

Participant questions were handled according to a predefined rule. Questions falling within the scope of the allocated script were answered using permitted wording; questions about the participant's own diagnosis, operative plan, anaesthetic technique or prognosis were referred to the responsible surgical or anaesthetic team; and any question about AI raised spontaneously by a participant in the attention‐control arm was acknowledged, recorded on the process form and deferred until after the endpoint assessment, so that AI‐specific content was not introduced into the comparator. No participant in either arm was refused an answer. Protocol deviations, randomisation deviations and allocation‐concealment records were logged prospectively and are reported in Supplementary Table D3.

Six nurses delivered the sessions, and each nurse delivered both conditions to an equal number of participants (10 per arm; Supplementary Table D4). Provider was therefore balanced across allocation groups by design. Provider was also included as a fixed categorical term in the process‐adjusted sensitivity model, and a random‐intercept model is reported in Supplementary Table H5. Fidelity monitoring relied on provider‐completed checklists and process records rather than independent observation, audio‐recording or a validated fidelity instrument. Accordingly, checklist completion documented whether the required components were covered but did not measure delivery quality. Intervention sessions were approximately 47 s longer on average than attention‐control sessions (6.01 ± 0.78 vs. 5.22 ± 0.66 min; SMD = 1.09), indicating incomplete time equivalence. Session tone, perceived clinician empathy and patient engagement were not measured, so experiential equivalence between the two conditions could not be established. These limitations are considered in Section 4.9.

### 2.10 Assessment Schedule and Outcome Hierarchy

Assessments were conducted at baseline before randomisation and communication delivery (T0), immediately after communication to capture initial response to the content (T1), and during the pre‐surgery waiting period after the post‐communication interval (T2). The primary outcome was T2 pre‐surgery anxiety measured using the APAIS anxiety subscale. The key secondary patient‐centred outcome was T2 perceived control. AI‐related uncertainty, information clarity and trust in the clinical team were measured at T1 as proximal communication‐uptake indicators. Exploratory analyses examined clinically meaningful anxiety reduction, anxiety trajectory and whether intervention effects differed by baseline eHealth literacy, AI familiarity or anxiety.

The following analyses were prespecified before database lock: the primary ANCOVA; the key secondary ANCOVA; the T1 comparisons of the three proximal indicators; the responder analysis at the ≥ 2‐point threshold, with the ≥ 1‐point threshold as a supportive check; the sensitivity analyses listed in Section 2.13; the mixed‐effects anxiety trajectory model; and exploratory moderation by baseline eHealth literacy, AI familiarity and baseline anxiety. Two sets of analyses were post hoc and are labelled as such wherever they appear: the subgroup displays by surgery type and anaesthesia type (Figure 3), which were prompted by the baseline imbalance subsequently observed on these variables; and the bias‐threshold and responder‐misclassification sensitivity analyses added during peer review (Section 3.6). All moderation, subgroup and pathway analyses are hypothesis‐generating, are reported without multiplicity adjustment and support no confirmatory claim.

### 2.11 Measures

#### 2.11.1 Primary Outcome: Pre‐Surgery Anxiety

Pre‐surgery anxiety was assessed using the Amsterdam Preoperative Anxiety and Information Scale anxiety subscale (APAIS; [35]). The anxiety subscale includes four five‐point items, yielding scores from 4 to 20, with higher scores indicating greater anxiety. The Turkish version has demonstrated acceptable reliability and validity [36]. The primary endpoint was T2 APAIS anxiety adjusted for baseline APAIS anxiety.

#### 2.11.2 Key Secondary Outcome: Perceived Control

Perceived control was measured using an 8‐item scale adapted for health care contexts and was prespecified as the key secondary outcome rather than as a covariate or predictor. Two considerations informed this decision. First, within the interpretive preparedness framework that guided the trial, perceived control is the cognitive appraisal the communication was designed to change: the protocol targeted orientation, predictability, understanding and the felt ability to ask questions, which is precisely the content of the scale. Entering it as a covariate would have adjusted away part of the hypothesised effect. Second, control‐appraisal and coping theory treats perceived control as a proximal consequence of the information and support a person receives rather than as a stable trait [17, 18], and preoperative preparation research has consistently modelled control as an outcome of psychological preparation [37]. Baseline perceived control was nevertheless measured at T0 and entered as the covariate in the ANCOVA model for the T2 score, so each participant serves as their own reference. The scale reflects patients' perceived ability to understand, orient themselves within and engage with care processes rather than control over surgical or anaesthetic decisions. Items were rated on a 5‐point scale, yielding scores from 8 to 40, with higher scores indicating greater perceived control. Internal consistency in the present sample was modest at each assessment point (Cronbach's α = 0.51 at T0, 0.66 at T1 and 0.60 at T2; Supplementary Table E1), and this endpoint is therefore interpreted supportively throughout.

#### 2.11.3 Proximal Communication‐Uptake Indicators

Three study‐specific four‐item indicators assessed the immediate response to the communication content: AI‐related uncertainty, information clarity and trust in the clinical team. Each was rated on a 5‐point scale, yielding scores from 4 to 20, with higher scores indicating more of the measured construct. Full item wording and response anchors, scoring rules, item‐level descriptive statistics, internal consistency and distributional behaviour are reported in Supplementary Appendix Sections C and E.

The three proximal indicators were developed for this trial and were not independently validated. Their content closely reflected the intervention components, which increases the likelihood that the measures captured recall and uptake of the delivered message. The attention‐control group did not receive the AI‐specific content addressed by these items, and the resulting group contrasts should therefore not be interpreted as independent clinical effects. Internal consistency was modest (Cronbach's α = 0.63 for AI‐related uncertainty, 0.72 for information clarity and 0.62 for trust), factor‐analytic, test–retest and convergent‐validity evidence was unavailable, and mild ceiling responding was present for information clarity (4.2%) and trust (3.3%). Throughout the article, these measures are therefore described as communication‐uptake indicators rather than independent patient‐reported outcome measures.

#### 2.11.4 Baseline Digital and Clinical Variables

Baseline eHealth literacy was assessed using the 8‐item eHealth Literacy Scale (eHEALS; [22]), with scores from 8 to 40 and higher scores indicating greater eHealth literacy. Turkish validation studies have reported acceptable psychometric properties [38, 39]. Baseline AI familiarity and internet health‐information seeking were measured using single self‐report items. Demographic and clinical variables included age, sex, previous surgery, chronic disease, education level, surgery type and anaesthesia type. Postoperative descriptors were collected for descriptive clinical context but were not used as covariates in the primary pre‐surgery endpoint model.

### 2.12 Scale Scoring and Data Quality

Multi‐item scale scores were recomputed from item‐level responses using predefined scoring rules; previously stored total‐score variables were not used for inference. Score ranges and directionality were verified before analysis, and internal consistency and distributional properties were examined descriptively. If item‐level missingness had occurred, scale scores were to be calculated when at least 80% of items were available using person‐mean prorating; otherwise, the scale score would be treated as missing. No primary endpoint data were missing, and no imputation was required for the primary analysis. These checks described measurement behaviour in the present sample and were not intended as full construct‐validation analyses, particularly for the study‐specific proximal communication‐uptake indicators. Full scoring rules, quality‐control documentation and psychometric/distributional summaries are provided in Supplementary Appendix Sections C and E.

### 2.13 Statistical Analysis

All analyses followed the intention‐to‐treat principle, with participants analysed in their allocated groups. Continuous variables were summarised as mean and standard deviation or median and interquartile range, and categorical variables as frequency and percentage. Baseline comparability was assessed descriptively using standardised mean differences rather than null‐hypothesis significance testing; values greater than 0.10 were considered potentially meaningful imbalances for sensitivity or exploratory analyses. The primary endpoint was analysed using baseline‐adjusted ANCOVA, with T2 APAIS anxiety as the dependent variable, treatment group as the fixed factor and T0 APAIS anxiety as the covariate. The primary estimand was the adjusted mean difference between groups. The key secondary endpoint, T2 perceived control, was analysed using an analogous baseline‐adjusted ANCOVA model. AI‐related uncertainty, information clarity and trust in the clinical team, measured at T1, were analysed as proximal communication‐uptake indicators using Welch‐corrected independent‐group tests with non‐parametric sensitivity checks. Clinically meaningful anxiety reduction was defined a priori as a decrease of at least 2 points on the APAIS anxiety subscale from T0 to T2; a 1‐point threshold was examined supportively. Groups were compared using absolute risk differences with 95% Newcombe confidence intervals and Fisher's exact tests, and number needed to treat was calculated where appropriate [40]. Sensitivity analyses included prognostic‐covariate adjustment, process adjustment, HC3 robust standard errors [41] and participant‐level bootstrap resampling with 5000 resamples. Anxiety trajectories were examined using mixed‐effects modelling, and exploratory moderation and subgroup analyses examined variation by baseline eHealth literacy, AI familiarity, baseline anxiety, surgery type and anaesthesia type. These analyses were considered supportive or hypothesis‐generating. Detailed diagnostics, model specifications and reproducibility procedures are reported in Supplementary Appendix Sections H–L. Statistical significance for the primary endpoint was set at a two‐sided alpha level of 0.05. Secondary, proximal and exploratory analyses were interpreted using effect sizes, 95% confidence intervals and clinical plausibility, without multiplicity adjustment. Analyses were performed using R (Version ≥ 4.3.0; [42]) and independently verified using IBM SPSS Statistics (Version 29).

Baseline covariate adjustment was prespecified in three layers. The primary model adjusted for the baseline value of the outcome alone. A prognostic model additionally adjusted for age, sex, AI familiarity, eHealth literacy and baseline outcome expectancy; expectancy was included specifically because it is the most plausible non‐specific mechanism in an unblinded behavioural trial. A process model additionally adjusted for session duration, the T1–T2 interval and provider code. Because participants were nested within six delivering nurses, a provider random‐intercept model was fitted as a clustering check (Supplementary Table H5).

Two additional post hoc sensitivity analyses assessed the robustness of the primary and responder findings. A bias‐threshold analysis quantified the magnitude of a systematic non‐specific response bias that would be required to reduce the adjusted anxiety difference below the prespecified clinically meaningful threshold or move the confidence interval to the null. A responder‐misclassification analysis examined increasingly conservative reclassification scenarios across both groups. These analyses were designed to assess how strongly plausible bias could alter the magnitude or direction of the findings; they do not remove the limitations of the unblinded design.

### 2.14 Analysis Population and Missing Data

The primary intention‐to‐treat population comprised all 120 randomised participants. All randomised participants completed the pre‐surgery endpoint assessment; therefore, no primary outcome data were missing, complete‐case and intention‐to‐treat analyses were identical for the primary endpoint, and no imputation was required. If outcome‐level missingness had exceeded 5% for secondary or exploratory outcomes, multiple imputation by chained equations was prespecified as a sensitivity approach under a missing‐at‐random assumption [43], but this was not required.

## 3 Results

### 3.1 Participant Flow and Baseline Characteristics

A total of 138 consecutive patients scheduled for elective surgery during the recruitment window were approached and assessed for eligibility; because no earlier screening filter was applied, this figure represents the complete consecutive series rather than a pre‐selected subset (Section 2.2). Eighteen patients were not randomised: 11 did not meet the eligibility criteria and 7 declined to participate. Reasons within the ineligible group, including cognitive, communication and clinical limitations and time‐critical operative circumstances, are summarised in Supplementary Table D1. The remaining 120 participants were randomised 1:1 to structured AI communication (*n* = 60) or attention‐control supportive communication (*n* = 60) (Figure 1). All randomised participants received their allocated condition and completed the baseline, immediate post‐communication and pre‐surgery endpoint assessments. No primary endpoint data were missing; the intention‐to‐treat and complete‐case populations were therefore identical for the primary analysis. As noted in Section 2.2, the absence of post‐randomisation attrition follows from the same‐day, pre‐surgery endpoint and the low participant burden rather than from any retention procedure, and should not be read as evidence of sustained engagement.

**Figure 1:**
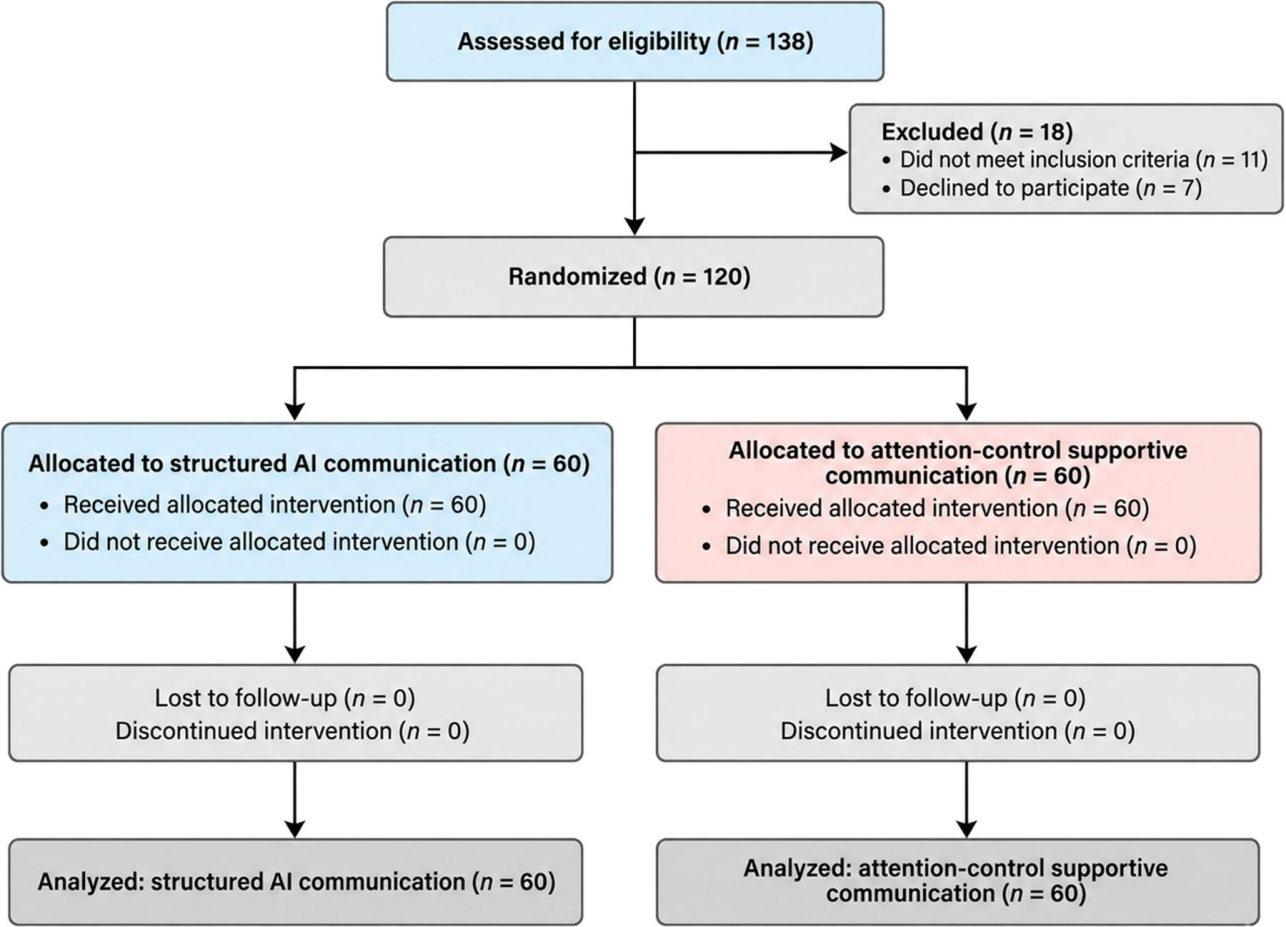
CONSORT flow diagram of participant recruitment, randomisation and follow‐up.

CONSORT flow diagram showing eligibility assessment, exclusions, randomisation, allocation to structured AI communication or attention‐control supportive communication, and completion of the baseline (T0), immediate post‐communication (T1) and pre‐surgery endpoint (T2) assessments. The 138 patients assessed for eligibility represent the complete consecutive series of adult elective surgery patients approached during the recruitment window; no earlier screening filter was applied. Reasons for ineligibility are summarised in Supplementary Table D1. All 120 randomised participants completed the primary endpoint assessment, which took place before surgery on the same day. Public and lived‐experience contribution to intervention development and interpretation is reported separately in Table 1 and Supplementary Appendix Section B.

Baseline demographic, clinical and digital‐literacy characteristics are presented in Table 2. Standardised mean differences (SMDs) are used descriptively rather than as significance tests. Several baseline and process variables exceeded the prespecified SMD threshold of 0.10, most notably baseline outcome expectancy (0.40), surgery type (0.32), anaesthesia type (0.30) and baseline APAIS anxiety (− 0.24); prognostic‐covariate and process‐adjusted sensitivity analyses were therefore retained to examine the robustness of the primary estimate, and each of these variables is addressed explicitly in Section 3.6. Extended baseline, clinical and process characteristics are provided in Supplementary Appendix Section D.

**Table 2:** Baseline characteristics by group.

Variable	Structured AI communication (*n* = 60)	Attention‐control supportive communication (*n* = 60)	SMD
Age, years	44.73 (12.60)	46.62 (14.35)	−0.14
Sex			0.10
Female	35 (58.3)	32 (53.3)	
Male	25 (41.7)	28 (46.7)	
Previous surgery			0.11
Yes	17 (28.3)	14 (23.3)	
No	43 (71.7)	46 (76.7)	
Chronic disease			0.05
Yes	9 (15.0)	8 (13.3)	
No	51 (85.0)	52 (86.7)	
Education level			0.24
Primary	16 (26.7)	22 (36.7)	
Secondary	18 (30.0)	19 (31.7)	
University	26 (43.3)	19 (31.7)	
Surgery type			0.32
General surgery	24 (40.0)	19 (31.7)	
Urology	15 (25.0)	19 (31.7)	
Orthopaedics	19 (31.7)	15 (25.0)	
Gynaecologist	2 (3.3)	7 (11.7)	
Anaesthesia type			0.30
General	35 (58.3)	26 (43.3)	
Regional	21 (35.0)	26 (43.3)	
Local/Sedation	4 (6.7)	8 (13.3)	
Expectancy score (1‐5)	3.49 (0.73)	3.20 (0.70)	0.40
AI familiarity (1‐5)	2.45 (0.89)	2.45 (0.93)	0.00
eHEALS T0	27.32 (4.58)	27.20 (4.87)	0.02
APAIS anxiety T0	11.62 (2.55)	12.27 (2.88)	−0.24
Perceived control T0	21.68 (3.84)	22.20 (3.86)	−0.13
VAS pain postop day 1	3.60 (1.61)	3.88 (1.51)	−0.18

*Note*. Continuous variables are mean (SD); categorical variables are *n* (%). For multi‐category variables, the displayed SMD is the maximum absolute category‐specific standardised difference. Extended baseline and process checks are reported in Supplementary Appendix Section D.

Abbreviations: APAIS, Amsterdam Preoperative Anxiety and Information Scale; eHEALS, eHealth Literacy Scale; SMD, standardised mean difference.

### 3.2 Primary Outcome: Pre‐Surgery Anxiety

At the T2 endpoint, unadjusted mean APAIS anxiety scores were 9.28 (SD = 2.55) in the structured AI communication group and 12.48 (SD = 3.01) in the attention‐control supportive communication group (Table 3). In the prespecified ANCOVA model adjusting for baseline (T0) anxiety, the adjusted mean difference (MD) was −2.56 points (95% CI, −2.87 to −2.26; F(1, 117) = 280.52; *p* < 0.001). This difference exceeded the prespecified minimum clinically important difference of 2 points and corresponded to a large unadjusted standardised between‐group effect at T2 (Cohen's d = −1.15 in the direction of lower anxiety with structured AI communication). The Shapiro–Wilk residual normality test was statistically significant (*p* = 0.014), whereas Levene's test did not show strong evidence of variance heterogeneity (*p* = 0.066) and the group × baseline anxiety interaction did not reject homogeneity of regression slopes (F = 1.78; *p* = 0.184). The same conclusion was retained using HC3 robust standard errors and participant‐level bootstrap intervals (Supplementary Appendix Sections F and H). Because the outcome was self‐reported by unblinded participants within the same clinical episode, the magnitude of this difference is interpreted in Sections 3.6 and 4.2 rather than taken at face value.

**Table 3:** Primary, secondary and proximal outcomes by group.

Outcome	Structured AI communication (*n* = 60)	Attention‐control supportive communication (*n* = 60)	Effect estimate (95% CI)	*p* value	Cohen's d
Primary: APAIS anxiety T2	9.28 (2.55)	12.48 (3.01)	Adjusted MD − 2.56 (− 2.87 to −2.26)	< 0.001	−1.15
Key secondary: Perceived control T2	25.83 (4.04)	22.62 (3.90)	Adjusted MD 3.73 (3.34 to 4.12)	< 0.001	0.81
Proximal: AI‐related uncertainty T1	7.33 (1.64)	11.70 (1.83)	Unadjusted MD − 4.37 (− 4.99 to −3.74)	< 0.001	−2.51
Proximal: Information clarity T1	17.20 (1.65)	12.80 (2.15)	Unadjusted MD 4.40 (3.71 to 5.09)	< 0.001	2.30
Proximal: Trust in clinical team T1	16.20 (1.71)	12.43 (2.13)	Unadjusted MD 3.77 (3.07 to 4.47)	< 0.001	1.95

*Note:* Adjusted MDs are from ANCOVA models adjusting for the baseline value of the relevant outcome for the primary and key secondary endpoints. The three proximal measures are study‐specific communication‐uptake indicators rather than validated clinical endpoints; their content mirrors the intervention script and the comparator arm was not exposed to that content, so their effect sizes are largely a design consequence and are not interpretable as clinical effects (Section 2.11.3). Proximal indicators were compared using Welch's *t*‐test with unadjusted mean differences; Mann–Whitney sensitivity results are reported in Supplementary Appendix Section F.

Abbreviations: APAIS, Amsterdam Preoperative Anxiety and Information Scale; MD, mean difference.

### 3.3 Key Secondary Outcome: Perceived Control

At T2, participants allocated to structured AI communication reported higher perceived control than those allocated to attention‐control supportive communication (25.83 ± 4.04 vs. 22.62 ± 3.90; adjusted MD, 3.73; 95% CI, 3.34 to 4.12; *p* < 0.001). The unadjusted standardised effect was large (Cohen's d = 0.81). Because internal consistency of the perceived control scale was modest at each assessment point (α = 0.51 to 0.66) and its residual normality and variance diagnostics were less favourable than those for the primary endpoint, this key secondary endpoint is interpreted supportively and alongside robust sensitivity information (Supplementary Appendix Sections E, F and H).

### 3.4 Proximal Communication‐Uptake Indicators (T1)

Immediately after the session, participants allocated to structured AI communication reported lower AI‐related uncertainty (7.33 ± 1.64 vs. 11.70 ± 1.83; unadjusted MD, −4.37; 95% CI, −4.99 to −3.74; *p* < 0.001; Cohen's d = −2.51), greater information clarity (17.20 ± 1.65 vs. 12.80 ± 2.15; MD, 4.40; 95% CI, 3.71 to 5.09; *p* < 0.001; d = 2.30) and greater trust in the clinical team (16.20 ± 1.71 vs. 12.43 ± 2.13; MD, 3.77; 95% CI, 3.07 to 4.47; *p* < 0.001; d = 1.95) than those allocated to attention‐control supportive communication (Table 3). Non‐parametric Mann–Whitney *U* tests confirmed each comparison (AI‐related uncertainty: *U* = 127.5, *p* < 0.001; information clarity: *U* = 3383.0, *p* < 0.001; trust: *U* = 3329.0, *p* < 0.001; Supplementary Appendix Section F). As set out in Section 2.11.3, these three measures index uptake of the intervention content rather than independent clinical outcomes. Their content closely mirrors the intervention script and the comparator arm was never exposed to that content, so effect sizes of this magnitude are largely a design consequence and are not interpreted as clinical effects.

### 3.5 Clinically Meaningful Anxiety Reduction

At the prespecified ≥ 2‐point reduction threshold, 48 of 60 participants (80.0%) allocated to structured AI communication were classified as responders, compared with 1 of 60 (1.7%) allocated to attention‐control supportive communication (Table 4). The absolute risk difference was 78.3% (95% CI, 64.5% to 86.6%; Fisher's exact *p* < 0.001), corresponding to a number needed to treat (NNT) of 1.28 (95% CI, 1.15 to 1.55). At the more liberal ≥ 1‐point threshold, 60 of 60 (100.0%) versus 8 of 60 (13.3%) were classified as responders (risk difference, 86.7%; 95% CI, 74.3% to 93.1%). Movement below the APAIS ≥ 11 anxiety threshold occurred in 21/40 (52.5%) versus 2/44 (4.5%) eligible participants. Extended responder rules and threshold analyses are provided in Supplementary Appendix Section G.

**Table 4:** Clinically meaningful anxiety response at T2.

Responder outcome	Structured AI communication	Attention‐control supportive communication	Risk difference (95% CI)	NNT (95% CI)	Fisher *p*
APAIS reduction ≥ 2 points	48/60 (80.0%)	1/60 (1.7%)	78.3% (64.5 to 86.6)	1.28 (1.15 to 1.55)	< 0.001
APAIS reduction ≥ 1 point	60/60 (100.0%)	8/60 (13.3%)	86.7% (74.3 to 93.1)	1.15 (1.07 to 1.35)	< 0.001
Moved below APAIS ≥ 11 threshold	21/40 (52.5%)	2/44 (4.5%)	48.0% (29.6 to 62.9)	2.09 (1.59 to 3.38)	< 0.001

*Note:* APAIS reduction = T0 − T2. Risk differences were calculated with Newcombe confidence intervals. NNT = number needed to treat, reported descriptively only: with a single responder in the attention‐control arm the risk ratio and NNT are arithmetically unstable and should not be read as efficacy estimates (Section 3.5). Inference is based on the continuous baseline‐adjusted mean difference. Extended responder, threshold and misclassification‐sensitivity analyses are reported in Supplementary Appendix Sections G and H.

These responder figures are extreme and warrant caution rather than emphasis, and three features account for much of their magnitude. First, the ≥ 2‐point threshold falls close to the centre of the intervention arm's change distribution (mean T0 → T2 change, −2.33) but far into the tail of the comparator arm's (mean change, +0.22), so a small shift in either distribution moves a large number of participants across the cut‐point; dichotomising a continuous outcome in this way discards information and produces unstable estimates [44]. Second, the near‐zero comparator rate makes the risk ratio, and therefore the NNT, arithmetically fragile: the entire contrast rests on a single comparator responder, and an NNT of 1.28 should not be read as a stable efficacy estimate. Third, and most importantly, an unblinded behavioural intervention delivered minutes before a self‐reported outcome is highly vulnerable to expectancy, novelty, social desirability and measurement‐reactivity effects, all of which act in the direction of the hypothesis [45]. We therefore report the responder analysis as a descriptive illustration of the continuous finding, base inference on the baseline‐adjusted mean difference, and quantify how much bias would be required to alter the conclusion in Section 3.6.

#### 3.5.1 Anxiety Trajectory Across Assessment Points

The linear mixed‐effects model showed a significant group‐by‐time pattern, with anxiety decreasing rapidly after structured AI communication and remaining lower at the pre‐surgery endpoint. Observed means showed that anxiety in the structured AI communication group decreased from T0 to T1 (− 3.20; 95% CI, −3.43 to −2.97; *p* < 0.001), increased modestly from T1 to T2 (+ 0.87; 95% CI, 0.54 to 1.20; *p* < 0.001) and remained lower than baseline at T2 (− 2.33; 95% CI, −2.57 to −2.10; *p* < 0.001). The attention‐control supportive communication group showed only small changes over time (T0–T1: +0.10; 95% CI, −0.10 to 0.30; T1–T2: +0.12; 95% CI, −0.15 to 0.38; T0–T2: +0.22; 95% CI, 0.03 to 0.41). Between‐group differences were evident at T1 (MD, −3.95; *p* < 0.001) and T2 (MD, −3.20; *p* < 0.001), indicating an early‐onset difference that persisted to the pre‐surgery endpoint. Because T2 fell within the same clinical episode, no inference about durability beyond that window is possible (Figure 2; Supplementary Appendix Section I).

**Figure 2:**
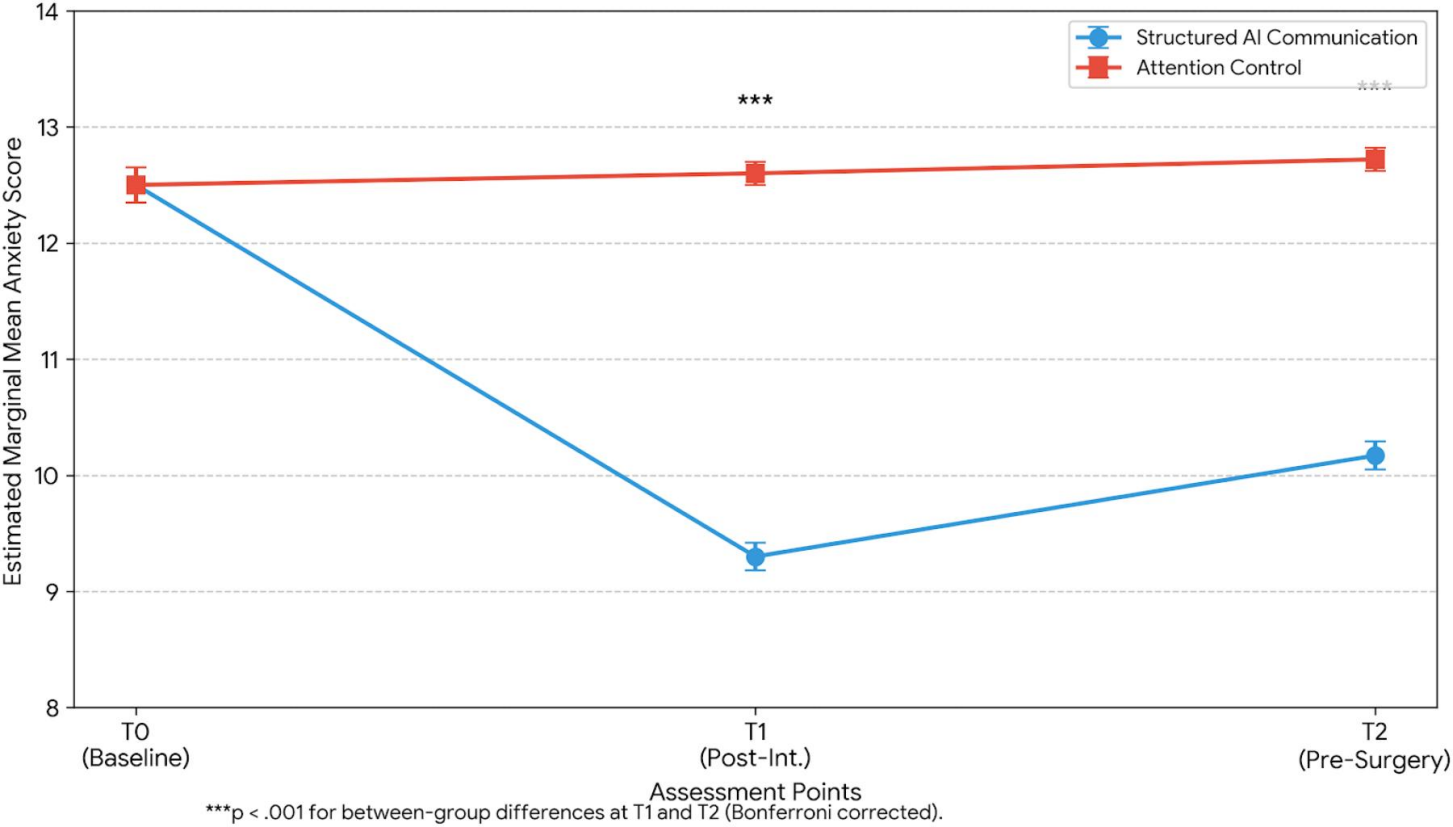
Trajectory of APAIS anxiety scores across assessment points. Data points represent estimated marginal means (± SE) from the linear mixed‐effects model. ***p < 0.001 for between‐group differences at T1 and T2 after Bonferroni correction.

### 3.6 Sensitivity Analyses

The primary finding was robust across all prespecified sensitivity analyses (Table 5). Adding age, sex, AI familiarity, eHealth literacy and baseline outcome expectancy to the ANCOVA model left the adjusted MD virtually unchanged (− 2.55; 95% CI, −2.85 to −2.25; *p* < 0.001; AIC = 296.7 vs. 299.5 for the primary model). This bears directly on the concern that expectancy generated the result: expectancy showed the largest baseline imbalance of any measured variable (SMD = 0.40), yet adjusting for it moved the estimate by 0.01 points. The process‐adjusted model incorporating session duration, T1–T2 interval and provider code yielded a similar, slightly larger estimate (− 2.71; 95% CI, −3.06 to −2.36; *p* < 0.001; AIC = 303.4), indicating that neither the imperfect time‐matching of the two conditions (mean session duration 6.01 vs. 5.22 min; SMD = 1.09) nor differences between the six delivering nurses accounts for the effect. A provider random‐intercept model gave a materially unchanged fixed effect of allocation (Supplementary Table H5). HC3 robust standard errors produced an adjusted MD of −2.56 (95% CI, −2.88 to −2.25; *p* < 0.001), and the participant‐level bootstrap interval ranged from −2.86 to −2.26 with negligible estimated bias (0.003).

**Table 5:** Sensitivity analyses for the primary outcome (APAIS T2).

Model	Adjusted MD	95% CI	p/diagnostic	AIC
Baseline‐adjusted ANCOVA	−2.56	−2.87 to −2.26	< 0.001	299.5
ANCOVA + prognostic covariates	−2.55	−2.85 to −2.25	< 0.001	296.7
Process‐adjusted model	−2.71	−3.06 to −2.36	< 0.001	303.4
HC3 robust SE	−2.56	−2.88 to −2.25	< 0.001	
Bootstrap	−2.56	−2.86 to −2.26	Bias 0.003	

*Note:* Prognostic covariates: age, sex, AI familiarity, eHealth literacy and baseline outcome expectancy. Process variables: session duration, T1–T2 interval and provider code. Bootstrap bias estimate: 0.003. All models shown were prespecified. The provider random‐intercept model, the post hoc bias‐threshold analysis and the post hoc responder‐misclassification analysis added during peer review are reported in Supplementary Tables H4 and H5. Full diagnostics are reported in Supplementary Appendix Section H.

Abbreviation: HC3, heteroscedasticity‐consistent robust standard errors.

In plain terms, the two groups were not perfectly matched at the start on outcome expectancy, surgery type, anaesthesia type and baseline anxiety, and the intervention sessions ran slightly longer than the comparator sessions. We therefore re‐ran the analysis several times, each time building one or more of these differences into the model, and asked whether the answer changed. It did not: in every model the intervention arm finished between roughly 2.3 and 3.1 APAIS points less anxious than the comparator arm. The exploratory strata in Figure 3 make the same point for surgery and anaesthesia type, where the effect is present within each category. Statistical adjustment cannot remove confounding by variables that were never measured, but it can show that the differences we did measure are not what produced the effect.

**Figure 3:**
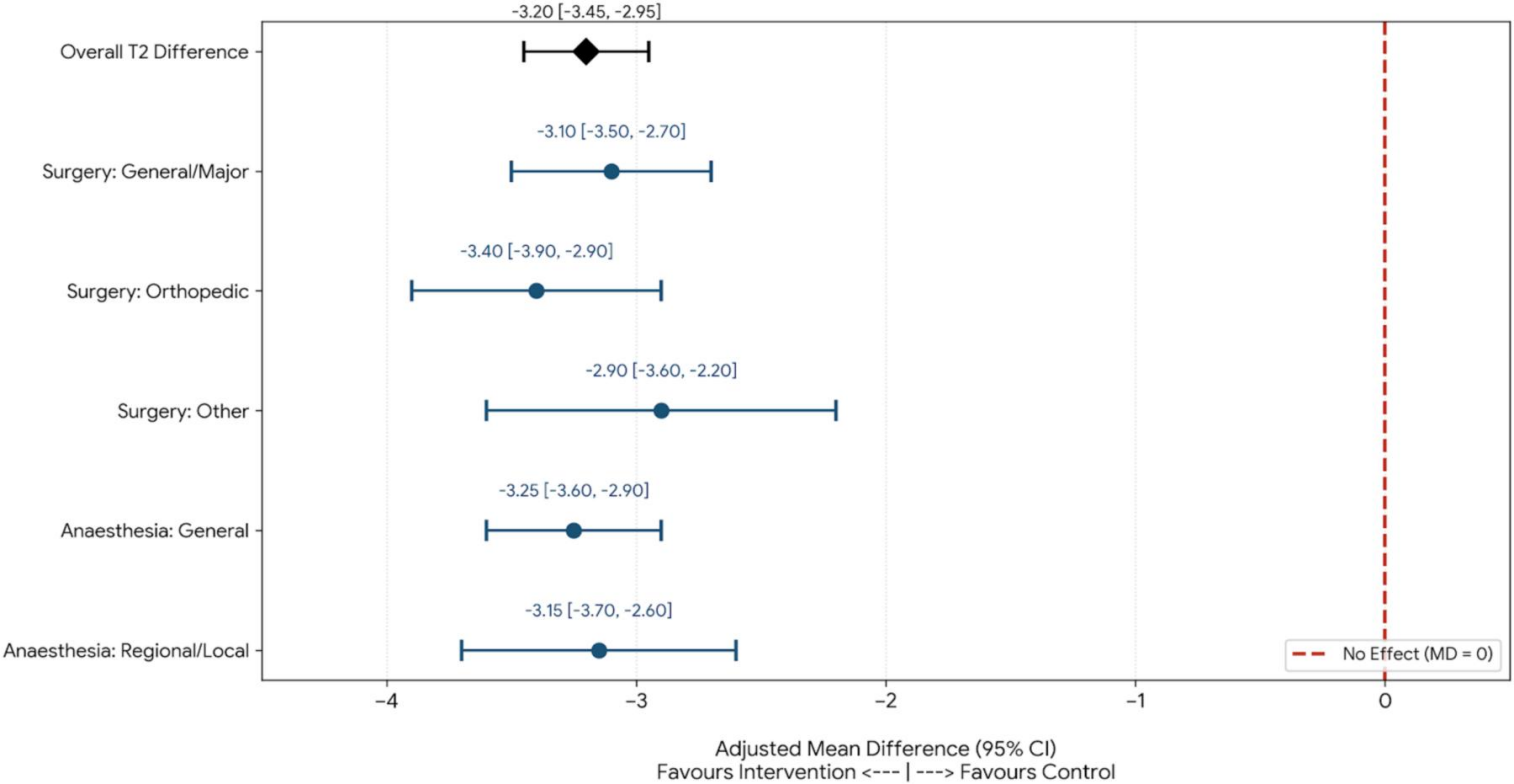
Forest plot of adjusted mean differences (with 95% CI) for APAIS T2 across surgery and anaesthesia subgroups. Estimates derived from separate ANCOVA models adjusting for baseline anxiety.

Two post hoc sensitivity analyses are reported in Supplementary Table H4. The bias‐threshold analysis indicated that a systematic non‐specific response bias favouring the intervention arm would need to reach 0.56 APAIS points (0.21 baseline SD) to reduce the adjusted mean difference below the 2‐point minimum clinically important difference, 2.26 points (0.83 SD) to move the upper confidence limit to the null, and 2.56 points (0.94 SD) to eliminate the point estimate. In the responder‐misclassification analysis, the risk difference remained 50.0% (95% CI, 33.8% to 62.6%) when 25% of intervention responders were treated as false positives and six comparator participants were reclassified as responders (36/60 vs. 6/60). Under the most conservative scenario examined (24/60 vs. 12/60), the risk difference was 20.0% (95% CI, 3.6% to 35.0%). The corresponding E‐values were 95.5 for the point estimate and 13.2 for the lower confidence limit. These analyses indicate that substantial bias would be required to reverse the direction of the finding, while remaining compatible with meaningful inflation of its magnitude.

### 3.7 Exploratory Moderation Analyses

Prespecified exploratory moderation suggested that the intervention effect on T2 APAIS anxiety varied by baseline eHealth literacy (interaction beta, 0.084; 95% CI, 0.022 to 0.146; p‐interaction = 0.008), with a larger estimated anxiety reduction among participants with lower eHealth literacy (low −1 SD: MD, −2.97; 95% CI, −3.38 to −2.55) and a smaller but still favourable estimate among those with higher eHealth literacy (high +1 SD: MD, −2.18; 95% CI, −2.59 to −1.77). No statistically clear interaction was observed for AI familiarity (*p* = 0.521) or baseline anxiety (*p* = 0.184). These moderation findings are hypothesis‐generating, were not adjusted for multiplicity and are reported with marginal estimates in Supplementary Appendix Section J.

### 3.8 Exploratory Subgroup Patterns

The subgroup displays by surgery type and anaesthesia type were post hoc, prompted by the baseline imbalance observed on these variables (Section 2.10). The direction and approximate magnitude of the intervention effect on T2 anxiety were consistent across both (Figure 3), with all point estimates favouring structured AI communication. These analyses were not powered for subgroup inference, several strata were small, no multiplicity adjustment was applied and they are interpreted descriptively only, as a check on whether the baseline imbalance could account for the primary result. Additional moderation safeguards and display details are provided in Supplementary Appendix Section J.

### 3.9 Exploratory Pathway‐Consistency Analysis

Because information clarity, trust and AI‐related uncertainty changed immediately after structured AI communication, we examined whether these proximal responses were consistent with the proposed interpretive‐preparedness pathway. Bootstrap estimates did not provide statistically precise evidence for separate indirect effects through information clarity (estimate, 0.17; 95% CI, −0.13 to 0.50) or trust in the clinical team (estimate, 0.06; 95% CI, −0.24 to 0.41). The direct effect after entering the pathway candidates remained large (− 2.80; 95% CI, −3.29 to −2.34), and the total baseline‐adjusted effect remained −2.56 (95% CI, −2.86 to −2.25). This analysis was exploratory and was not designed as a confirmatory mediation test; the results indicate only that anxiety reduction was accompanied by change in the intended communication targets, and they establish no causal pathway (Supplementary Appendix Section K).

### 3.10 Adverse Events

No adverse events, unintended harms or protocol‐related safety concerns were reported in either group. No participant discontinued the allocated communication condition because of distress or discomfort.

## 4 Discussion

### 4.1 Principal Findings

In this attention‐controlled randomised trial, a brief, public‐informed protocol of structured AI communication was associated with lower immediate pre‐surgery anxiety, higher perceived control and higher proximal indicators of communication uptake than time‐matched attention‐control supportive communication. The primary anxiety finding was statistically robust across every prespecified sensitivity analysis and exceeded the prespecified minimum clinically important difference, with a baseline‐adjusted mean difference of −2.56 points. Clinically meaningful anxiety reduction was also more frequent after structured AI communication; as set out in Sections 3.5 and 3.6, that responder contrast is unstable and is reported descriptively. Three boundaries frame everything that follows. The trial did not evaluate an AI system, diagnostic algorithm or decision‐support tool, so it is not evidence about AI technology; it tested whether brief communication about AI‐related information, uncertainty and professional accountability could support patients' interpretive preparedness. All outcomes were self‐reported by unblinded participants within a single pre‐surgery window, so the findings describe immediate self‐report and not durable change. And the study was conducted in one hospital with 120 patients, so the estimates are provisional. Within those boundaries, the results suggest that responsible‐AI principles can be translated into patient‐facing perioperative communication, and public and lived‐experience contribution was central to that translation, helping ensure the protocol addressed the questions patients actually bring to technology‐enabled care: what AI can and cannot do, who remains accountable and how general AI‐related information applies to an individual.

### 4.2 Interpretation of the Primary Anxiety Reduction

The anxiety difference observed in this trial was larger than effects generally reported for communication‐focused or psychological preparation interventions in preoperative care. Meta‐analytic evidence typically indicates small‐to‐moderate or moderate effects, below the unadjusted T2 standardised difference observed here [37, 46]. One possible explanation is that the protocol addressed a specific and emerging concern involving AI‐related information, uncertainty and professional accountability that is not routinely covered in preoperative education. It clarified the limits of AI‐related information, professional responsibility and the need for clinician‐led contextualisation. Methodological factors are also likely to have contributed. The intervention was brief, behavioural and unblinded, was delivered by a nurse who knew the allocation, and was followed shortly by participant self‐report in the same clinical episode. Expectancy, novelty, social desirability and measurement reactivity can all increase apparent intervention effects under these conditions [45]. The magnitude should therefore be interpreted cautiously pending independent replication.

Three pieces of evidence bear on how far the second explanation can account for the finding, and none of them is decisive. Baseline outcome expectancy was measured, showed the largest baseline imbalance of any variable (SMD = 0.40), and adjusting for it changed the estimate by 0.01 points, which is difficult to reconcile with expectancy as the principal driver. The bias‐threshold analysis indicates that a systematic non‐specific bias of 0.56 APAIS points (0.21 baseline SD) would be needed to bring the estimate below the minimum clinically important difference and 2.56 points (0.94 SD) to abolish it altogether; the first of these is entirely plausible for an unblinded self‐report measure, while the second is not. And the responder‐misclassification analysis shows the direction of the finding surviving deliberately extreme assumptions. Our reading is therefore that the direction of the effect is unlikely to be an artefact, while the magnitude is probably inflated, and that the true effect under blinded conditions would most plausibly sit somewhere between the minimum clinically important difference and the observed estimate. The responder analysis is consistent with the continuous outcome but adds little independent information: 80.0% of the structured AI communication group met the prespecified ≥ 2‐point threshold against 1.7% of the comparator group, and with a single comparator responder the resulting NNT of 1.28 is arithmetically fragile rather than clinically informative. The continuous baseline‐adjusted analysis remains the stable basis for inference, and only a blinded or longer‐term replication can settle the magnitude.

### 4.3 Perceived Control and the Interpretive Preparedness Framework

The increase in perceived control (adjusted MD, 3.73; unadjusted d = 0.81) is consistent with the distinction between anxiety as an emotional state and perceived control as a cognitive appraisal relating to orientation, predictability and the ability to ask questions within care [17, 18]. Within the interpretive preparedness framework, patients do not need to control surgical or anaesthetic decisions; they may benefit from understanding what AI‐related information can and cannot mean, who remains accountable and how general information should be contextualised for their own care. Public advisory feedback supported this reading, emphasising that patients value knowing where responsibility lies. The finding also aligns with uncertainty communication literature suggesting that effective communication does not eliminate uncertainty but helps patients understand what is known, what remains uncertain and who will guide interpretation and next steps [15, 16], and with patient‐centred care principles in which understanding, orientation and opportunities to ask questions are central to engagement [26, 47]. Two cautions apply. Internal consistency of the perceived control scale was modest at every assessment point (α = 0.51 to 0.66), so the measure itself is imprecise; and the endpoint was secondary and assessed within an immediate pre‐surgery window. This result should therefore be treated as supportive evidence for the interpretive preparedness framework, not as evidence of durable empowerment.

### 4.4 Reduction in AI‐Related Uncertainty and Calibrated Trust

A key proximal communication‐uptake finding was that AI‐related uncertainty was lower after structured AI communication than after attention‐control supportive communication (MD = −4.37, 95% CI − 4.99 to −3.74, *p* < 0.001, d = −2.51), alongside large improvements in information clarity and trust in the clinical team. This pattern is consistent with uncertainty communication frameworks that distinguish uncertainty management from simple reassurance [14, 15]. The intervention did not present AI‐related information as definitive; instead, it acknowledged that such information may be general, probabilistic or uncertain while clarifying professional responsibility and individual clinical interpretation. Public contributors supported this framing by emphasising that “uncertainty” may be anxiety‐provoking when left unexplained, whereas linking uncertainty with professional accountability, human oversight and calibrated trust was perceived as more reassuring and useful. Thus, lower AI‐related uncertainty in this trial likely reflected improved understanding of the role, limits and oversight of AI‐related information rather than simple reassurance about AI. This pattern is consistent with communication research in other uncertainty‐laden contexts, including genetic risk, serious illness and cancer care, where patients may benefit when clinicians help them interpret probabilistic information and clarify what is known, what remains uncertain and who will guide next steps [48, 49]. The present trial extends this logic to AI‐related health communication: clinicians should neither overpromise certainty nor leave AI‐related uncertainty unframed, but should help patients interpret such information within an accountable, clinician‐led framework. Because the AI‐related uncertainty, information clarity and trust measures were study‐specific and closely aligned with the intervention content, these findings should be interpreted as proximal indicators of communication uptake rather than as independently validated clinical endpoints.

The reduction in AI‐related uncertainty also permits an alternative interpretation. Lower reported uncertainty may indicate better understanding of the role, limits and oversight of AI‐related information, but it could also reflect misplaced certainty if participants inferred that AI was definitive, authoritative or institutionally validated. The item set measured felt uncertainty rather than the accuracy of underlying beliefs and therefore could not distinguish calibrated understanding from overconfidence. Information clarity increased alongside the reduction in uncertainty, and the trust items referred to the clinical team rather than to AI, which is consistent with the intended interpretation but does not establish it. Future studies should incorporate measures of belief accuracy and trust calibration. In the present study, the finding is therefore described as reduced felt uncertainty rather than demonstrated calibrated trust.

### 4.5 Exploratory Mechanistic, Literacy and Subgroup Findings

Exploratory pathway‐consistency analyses offered limited but conceptually useful support for the proposed interpretive‐preparedness mechanism. Information clarity and trust in the clinical team improved substantially after structured AI communication, and the total baseline‐adjusted anxiety difference remained large. However, bootstrap confidence intervals for the separate pathway estimates through clarity and trust crossed zero; these findings should therefore not be interpreted as confirmatory mediation, but as evidence that anxiety reduction was accompanied by proximal changes in the intended communication targets. This interpretation is consistent with patient‐centred communication literature emphasising clinician trust, shared understanding and relational accountability in supporting patient engagement and preparedness [23, 25]. In AI‐related communication, trust should be understood as trust in accountable clinician‐led interpretation rather than uncritical trust in AI itself.

Exploratory moderation suggested that baseline eHealth literacy may have influenced the magnitude of the intervention effect, with larger estimated anxiety reduction among participants with lower baseline eHealth literacy and smaller, but still favourable, effects among those with higher eHealth literacy. This pattern is clinically plausible because patients with lower eHealth literacy may face greater difficulty locating, appraising and applying digital or AI‐related health information [20, 21]. AI familiarity and baseline anxiety did not show statistically clear moderation. Subgroup displays also showed directionally consistent effects across surgery and anaesthesia types, with all point estimates favouring structured AI communication. However, moderation and subgroup analyses were exploratory, not powered for definitive inference and not adjusted for multiplicity. Larger multisite studies should test whether clarity, clinician‐led trust and reduced AI‐related uncertainty act as mechanisms, and whether patients with lower digital‐literacy needs, lower baseline trust or higher‐risk surgery derive differential benefit from AI‐related communication.

### 4.6 Comparison With the General AI Attitude Literature

This trial adds interventional evidence to a field largely shaped by cross‐sectional surveys, qualitative studies and conceptual work on public and patient attitudes toward AI in healthcare. Existing studies indicate that patients often worry about clinician replacement, weakened professional accountability and difficulty interpreting AI‐generated or AI‐supported information [7, 9]. Recent qualitative evidence further suggests that public judgements about AI are relational rather than purely technical, depending on perceived caregiving relationships, ethical oversight, professional responsibility and the continuing role of clinicians [50, 51]. The present trial extends this literature by testing whether brief structured clarification about AI‐related information, uncertainty, human oversight and professional accountability can improve immediate patient‐centred outcomes in a perioperative setting. The findings suggest that responsible‐AI principles such as transparency, accountability and human oversight can be translated into short, patient‐facing explanations that support interpretive preparedness before surgery. This supports the view that patient‐centred AI implementation should treat communication as a core component of technology‐enabled care, not as an optional add‐on after technical deployment [1, 5].

### 4.7 Governance, Data Privacy and the Boundaries of Bedside AI Communication

Communicating with patients about AI sits inside a wider system of digital‐health governance that this trial did not test. Whether a patient can meaningfully act on a statement such as “clinical decisions remain the responsibility of your care team” depends on arrangements that are invisible at the bedside: how health data are collected, stored, linked and shared; which organisation carries accountability when an AI‐supported process contributes to an error; how consent for secondary data use is obtained; and what standards govern the provenance and quality of the data on which patient‐facing tools are built. Recent work on the governance of medical data as a health‐system asset sets out how fragmented these arrangements remain across jurisdictions, and how much depends on institutional reform, ethical frameworks and interoperability standards rather than on clinician communication [52]. Public advisors in the present study raised data privacy spontaneously during the post‐analysis discussion, which suggests patients recognise this dependency even when it is not part of the conversation.

Two practical boundaries follow from this analysis, although neither was tested empirically. First, communication can explain existing accountability arrangements but cannot compensate for weak governance. Clear communication may otherwise generate trust that institutional systems have not earned. Second, the protocol did not address data privacy, secondary data use or detailed organisational accountability because these issues could not be explained accurately and consistently within a brief preoperative conversation. The study therefore provides no evidence about how patients would respond to such content or where the optimal boundary of bedside communication lies. Future work should determine which information belongs in clinician‐patient discussion, written materials and institutional disclosure, ideally through co‐production with patients and the public [30, 31].

### 4.8 PPIE Contribution and Its Impact

Patient and public involvement and engagement strengthened this study because AI‐related communication is not only technical or clinical, but also interpretive and relational. Three lived‐experience advisors reviewed the patient‐facing materials during protocol refinement, helping reduce technical or promotional wording and strengthen messages about professional accountability, human oversight and individual clinical contextualisation. After the primary analysis, a public advisory group reviewed a plain‐language summary of the findings and informed the final interpretation, including the framing of calibrated trust, the patient‐level meaning of anxiety reduction and the need for concise practitioner‐facing recommendations. This contribution improved the relevance, acceptability and patient‐facing clarity of the communication protocol, while remaining consultative rather than fully co‐produced. Public contributors did not share governance authority over trial design, outcome selection, recruitment, randomisation, analysis or dissemination, and they did not alter predefined outcomes or the analysis plan. GRIPP2‐mapped documentation in Supplementary Appendix Section B reports the timing, scope and influence of public contribution and may inform future patient‐centred communication trials involving AI‐related health information [53].

### 4.9 Strengths and Limitations

This study has several strengths. The randomised, attention‐controlled design strengthens causal interpretation by comparing structured AI communication with time‐matched attention‐control supportive communication rather than with usual care alone. The sample size was prospectively determined for the primary endpoint, the recruitment target was met and every randomised participant completed the endpoint assessment. The intervention was brief, standardised and feasible for routine perioperative settings, with scripts, wording boundaries and delivery checklists supplied to support replication. Analyses were prespecified, adjustment layers were defined in advance, and the trial addressed a communication gap that standard preoperative preparation does not cover.

Several limitations should be considered, and they constrain the claims that can be made. The single‐centre design and the recruitment of adult elective surgery patients in one tertiary hospital limit generalisability across health systems, languages, surgical populations and cultural contexts; the results should not be extrapolated to emergency surgery, paediatric care, non‐surgical settings or health systems with different patterns of AI deployment and public discourse. All endpoints were assessed within a single pre‐surgery window, so the trial provides no information about durability: the design cannot distinguish a lasting change in interpretive preparedness from a transient response to a recent conversation, and no postoperative, behavioural or clinical outcome was measured. Statements about anxiety reduction in this article therefore refer to immediate, self‐reported differences only.

Participants and providers could not be blinded. The attention‐control design, self‐administered questionnaires, coded analysis labels and sensitivity analyses reduced some sources of bias, but expectancy, novelty, social desirability and measurement reactivity remain plausible contributors to the observed magnitude [45]. Intervention sessions were approximately 47 s longer on average than comparator sessions (SMD = 1.09), so the conditions differed slightly in contact time as well as content, although adjustment for duration did not attenuate the estimate. Delivery was not independently observed, audio‐recorded or rated using a validated fidelity instrument; provider‐completed checklists documented component coverage rather than delivery quality. Session tone, perceived clinician empathy and patient engagement were not measured, so experiential equivalence between conditions was not demonstrated. Six nurses delivered equal numbers of sessions in both groups, reducing provider confounding by design. Nurse‐level clustering was addressed mainly as a fixed effect, and residual clustering may have produced confidence intervals that were slightly too narrow.

Several baseline and process descriptors exceeded the prespecified SMD threshold of 0.10, most notably outcome expectancy (0.40), surgery type (0.32) and anaesthesia type (0.30). These were addressed through prognostic‐covariate and process‐adjusted sensitivity analyses and through the exploratory strata in Figure 3, but residual confounding by unmeasured variables remains possible. The proximal communication‐uptake indicators were study‐specific, unvalidated, of modest internal consistency (α = 0.62 to 0.72) and closely aligned with the intervention script, and should be read only as evidence that the intended message was received. Baseline internal consistency of the APAIS anxiety subscale was below the conventional threshold (α = 0.54), although reliability was acceptable at T1 and T2, and internal consistency of the perceived control scale was modest at each time point (α = 0.51 to 0.66); both endpoints were therefore interpreted alongside baseline‐adjusted modelling and robust sensitivity analyses. Subgroup, moderation and pathway analyses were exploratory, were not powered for inference and were not adjusted for multiplicity, and the pathway analysis is explicitly not a confirmatory mediation test.

The intervention format also limits generalisation to other forms of AI communication. The protocol was a brief, scripted and predominantly one‐way clinician‐delivered explanation. By contrast, patients may encounter conversational and generative AI through chatbots, symptom checkers and search tools that allow repeated questioning and provide fluent responses without real‐time clinician contextualisation. The present trial did not evaluate those interactions and should not be interpreted as evidence about communication needs or outcomes when patients use such tools independently.

Finally, no AI system was used in the participants' own care, so these findings are not evidence that AI technology improves patient outcomes. Public contribution was structured and documented across protocol refinement and post‐analysis interpretation but remained consultative rather than co‐produced: contributors did not share governance authority and were not involved in recruitment, randomisation, outcome measurement, analysis or dissemination planning. Future studies should strengthen PPIE through co‐design workshops, cognitive interviews, more diverse advisory panels and public involvement in dissemination planning.

### 4.10 Clinical Implications

These findings suggest, provisionally, a direction for perioperative practice and patient‐centred communication about technology‐enabled care. A brief, script‐supported conversation similar to the protocol tested here may help clinicians address patients' questions about AI‐related information without requiring specialist technical training. The aim is not to promote AI but to clarify how AI‐related information should be understood within accountable, clinician‐led care. The practitioner points refined with public input offer concise guidance: explain that AI‐related information may support care but does not replace professional clinical judgement; state who remains responsible for decisions and how general AI‐related information is interpreted for the individual patient; and invite questions about AI, uncertainty, accountability and human oversight using brief, non‐technical language. Because the evidence base is a single unblinded trial with immediate self‐reported endpoints, we would frame this as a communication practice worth evaluating locally rather than as an intervention ready for implementation at scale. Durability, transferability across settings and effects on any outcome beyond immediate self‐report all remain untested.

### 4.11 Future Research

Several priorities follow from these limitations. Multicentre trials should determine whether the immediate effects persist beyond the pre‐surgery period and influence postoperative recovery, patient experience, satisfaction or longer‐term trust. Future trials should incorporate independent fidelity assessment through audio‐recording or observer rating and measure perceived clinician empathy, session tone and patient engagement in both groups. Separating outcome assessment from intervention delivery in time and personnel would further reduce measurement reactivity. Measures of belief accuracy and trust calibration are needed to distinguish reduced felt uncertainty from misplaced certainty. Adapted formats, including video explanations, written materials, digital information sheets and clinician‐led scripts, should be evaluated across diverse clinical and non‐surgical settings. Research should also address conversational and generative AI, where patients set the agenda and no clinician may be present, and should test communication protocols alongside actual AI decision‐support tools. Co‐design workshops, cognitive interviews and diverse advisory panels are needed to refine the protocol for different digital‐literacy levels, cultural backgrounds and informational needs. Implementation research should identify feasible and acceptable strategies for integration into routine workflows.

## 5 Conclusion

In a single‐centre randomised trial, a brief, public‐informed protocol of structured AI communication was associated with lower immediate pre‐surgery anxiety and higher perceived control than time‐matched attention‐control supportive communication among adult elective surgery patients. Because the intervention was unblinded and every endpoint was self‐reported within the pre‐surgery window, the magnitude of these differences is likely inflated and their durability is unknown. The trial supports patient‐centred communication about AI‐related information as a candidate component of interpretive preparedness in technology‐enabled care; it does not demonstrate that AI technology improves clinical outcomes, and it does not establish benefit beyond immediate self‐report. Multicentre, co‐designed studies with independent fidelity assessment and longer follow‐up are needed to determine durability, implementation and patient experience across health systems.

## Author Contributions

**Volkan Gökmen:** supervision, data curation, writing – review and editing, validation, methodology, conceptualisation, investigation, funding acquisition, project administration, resources, writing – original draft. **Burcu Demir Gökmen:** supervision, data curation, writing – review and editing, validation, methodology, conceptualisation, investigation, funding acquisition, project administration, resources, writing – original draft.

## Funding

The authors have nothing to report.

## Ethics Statement

Ethical approval was obtained from the Ağrı İbrahim Çeçen University Scientific Research Ethics Committee (Decision No. 56, approval date: 29 January 2026; document no. E‐95531838‐050.99‐164656, dated 30 January 2026). All participants provided written informed consent before enrolment. The study was conducted in accordance with the Declaration of Helsinki and reported in accordance with the CONSORT guidelines for randomised trials.

## Conflicts of Interest

The authors declare no conflicts of interest.

## Declaration of Use of AI‐Assisted Tools

During manuscript preparation, ChatGPT (OpenAI) was used solely to assist with text and language editing. The authors critically reviewed, verified and approved all content and take full responsibility for the integrity, accuracy and originality of the work.

## Supporting information


Supporting File

## Data Availability

The data that support the findings of this study are available on request from the corresponding author. The data are not publicly available due to privacy or ethical restrictions. De‐identified participant data, statistical code and study materials are available from the corresponding author upon reasonable request after publication, subject to institutional and ethical regulations.
