# Supplementary material for: Patient‐Centred Communication About AI‐Related Information in Perioperative Care: An Attention‐Controlled Randomised Trial of Anxiety, Perceived Control and Interpretive Preparedness
Source: Health Expect. 2026 Nov 13;29(6):e70838. doi: 10.1111/hex.70838 (PMC13761288; doi:10.1111/hex.70838)
Supplement: Supplementary file 1 [file HEX-29-e70838-s001.docx]

**Supplementary Appendix S1**

Intervention Materials, Public Contribution and Extended Analyses

Patient-Centred Communication About AI-Related Information in Perioperative Care: An Attention-Controlled Randomized Trial of Anxiety, Perceived Control and Interpretive Preparedness

This supplementary appendix provides intervention materials, patient and public contribution documentation, scoring procedures and extended analyses supporting the main manuscript.

**Supplementary Appendix Contents**

| **Section** | **Content** |
| --- | --- |
| A | Communication protocols and delivery checklist |
| B | Patient and public contribution documentation |
| C | Outcome measures, item-level scoring and data quality procedures |
| D | Extended baseline, clinical and process characteristics |
| E | Psychometric and distributional analyses |
| F | Extended primary, secondary and proximal outcome analyses |
| G | Clinically meaningful response and threshold analyses |
| H | Sensitivity analyses and model diagnostics |
| I | Mixed-effects anxiety trajectory analysis |
| J | Exploratory moderation analyses |
| K | Exploratory pathway-consistency and mediation analyses |
| L | Reporting checklist mapping and reproducibility materials |

# **Section A. Communication Protocols and Delivery Checklist**

This section provides the structured AI communication script, the attention-control supportive communication script and delivery checklist used to support standardisation. The scripts are reported as English-language manuscript versions of the patient-facing content. The intervention clarified the role, limits and oversight of AI-related information and did not imply that an AI system was making decisions about the participant’s own surgery.

**Supplementary Table A1. Structured AI communication script**

| **Component** | **Patient-facing wording** |
| --- | --- |
| Opening statement | Before your operation, I would like to briefly explain how artificial intelligence-related health information should be understood. This does not mean that an AI system is making decisions about your surgery. The purpose is to help you understand general AI-related information and how it fits within clinician-led care. |
| AI as supportive, not substitutive | Artificial intelligence may support health professionals by helping with information, patterns or workflow. It does not replace doctors, nurses or other members of the clinical team. |
| Professional human oversight | Clinical decisions remain the responsibility of qualified health professionals. Your care is interpreted and managed by your clinical team according to your own condition, history and surgical plan. |
| AI-related uncertainty | AI-related information may be general, probabilistic or based on patterns from many people. Such information is not automatically a final answer for an individual patient. |
| Contextualisation | Information found online or described as AI-supported may not directly apply to your own situation. Your clinical team interprets any relevant information in the context of your own care. |
| Invitation to questions | Please ask any question that is unclear, including questions about AI, uncertainty, accountability, your operation or who is responsible for decisions. |
| Closing statement | The main point is that technology may support care, but your clinical team remains responsible for interpreting information and caring for you as an individual patient. |

Note. Flexible wording was permitted only when the core meaning of each component was preserved.

**Supplementary Table A2. Attention-control supportive communication script**

| **Component** | **Patient-facing wording** |
| --- | --- |
| Opening statement | Before your operation, I would like to briefly check whether you have any general questions about the routine preparation process. |
| General supportive clarification | The team will continue to support you before the operation. You can ask questions about preparation, timing and what will happen next. |
| Routine perioperative reassurance | Your care will be provided according to the usual hospital procedures and the clinical team will continue to monitor you. |
| Opportunity to ask questions | Please ask if anything about the routine preparation process is unclear. |
| Closing statement | The aim of this conversation is to make sure you feel supported and know that you can ask questions before surgery. |

Note. The comparator was time-matched and supportive but excluded AI-specific content, explanations of AI-related uncertainty, AI oversight, AI replacement concerns and AI contextualisation.

**Supplementary Table A3. Permitted flexible wording and prohibited content**

| **Domain** | **Permitted wording** | **Prohibited wording** |
| --- | --- | --- |
| AI role | AI may support health professionals. | AI will decide your care. |
| Human oversight | Clinicians remain responsible for interpretation and decisions. | The computer or AI is responsible for decisions. |
| Uncertainty | AI-related information may be general or probabilistic. | AI is always accurate or definitive. |
| Contextualisation | Your clinical team interprets information for your own case. | Online or general AI advice applies directly to you. |
| Trust | The goal is to understand the role and limits of AI within clinician-led care. | The goal is to make you trust AI. |

**Supplementary Table A4. Checklist-guided delivery form**

| **Checklist item** | **Response** | **Notes** |
| --- | --- | --- |
| Supportive role of AI clarified | Yes / No |  |
| Professional human oversight clarified | Yes / No |  |
| AI-related uncertainty explained | Yes / No |  |
| General versus patient-specific information explained | Yes / No |  |
| Questions invited | Yes / No |  |
| No claim that AI was used to make the participant’s own surgical decision | Yes / No |  |
| Session duration recorded | Minutes |  |
| Provider code recorded | Provider ID |  |

Note. This checklist supported standardised delivery and process documentation. It was not an independent observational fidelity rating.

**Supplementary Table A5. TIDieR description of the two study conditions**

| **TIDieR item** | **Structured AI communication (intervention)** | **Attention-control supportive communication (comparator)** |
| --- | --- | --- |
| 1. Brief name | Structured AI communication | Attention-control supportive communication |
| 2. Why (rationale, theory) | Interpretive preparedness framework; responsible-AI principles (transparency, accountability, human oversight) translated into brief patient-facing language. | Controls for the non-specific effects of nurse attention, interpersonal contact, communication time and the opportunity to ask questions. |
| 3. What (materials) | Written script (Table A1); permitted and prohibited wording list (Table A3); checklist-guided delivery form (Table A4). | Written script (Table A2); the same checklist-guided delivery form (Table A4). |
| 4. What (procedures) | Five predefined components: AI as supportive not substitutive; professional human oversight; continuing clinical responsibility; uncertainty and generalisability of AI-related information; contextualisation to the patient's own situation. Opening statement explicitly denies that any AI system is making decisions about the participant's surgery. | Supportive clarification about routine perioperative preparation, timing and next steps, with an explicit invitation to ask questions. No AI-specific content of any kind. |
| 5. Who provided | Six registered nurses who completed a structured orientation covering protocol components, patient-centred language, permitted and prohibited wording, time equivalence and the question-handling rule. | The same six nurses; each delivered both conditions to 10 participants per arm. |
| 6. How | Face-to-face, individual, verbal delivery. | Face-to-face, individual, verbal delivery. |
| 7. Where | Preoperative preparation area of a single tertiary hospital. | Identical setting. |
| 8. When and how much | One session before surgery; 5–7 minutes planned; delivered mean 6.01 minutes (SD 0.78). | One session before surgery; time-matched; delivered mean 5.22 minutes (SD 0.66). Time-matching was therefore imperfect (SMD = 1.09). |
| 9. Tailoring | Flexible wording permitted within predefined boundaries provided each component's core meaning was preserved. Questions within script scope answered using permitted wording; clinical questions referred to the surgical or anaesthetic team. | Same question-handling rule. Any spontaneous participant question about AI was acknowledged, recorded and deferred until after the endpoint assessment. |
| 10. Modifications | None after trial commencement. | None after trial commencement. |
| 11. How well (planned) | Written script; checklist-guided delivery; recording of session duration, provider code and item-by-item checklist completion. | Identical monitoring procedures. |
| 12. How well (actual) | All checklist items were recorded as completed for all sessions. Delivery was not independently observed, audio-recorded or rated on a validated fidelity instrument, and no measure of tone, perceived empathy or engagement was obtained. | As for the intervention arm. Comparability of the two conditions on tone, perceived empathy and patient engagement was assumed by design but not measured. |

Note. Prepared in accordance with the Template for Intervention Description and Replication (TIDieR) checklist (Hoffmann et al., 2014) in response to peer review. Item 12 records that delivery monitoring consisted of self-completed checklists and process forms only: no independent observation, audio-recording or validated fidelity rating was undertaken, and no measure of session tone, perceived clinician empathy or patient engagement was obtained in either arm.

# **Section B. Patient and Public Advisory Group Documentation**

This section provides the consultative advisor and patient/public advisory documentation supporting patient-facing interpretation of the communication protocol and study findings. The public contribution component strengthened clarity, acceptability, patient relevance and manuscript presentation, but did not alter recruitment, randomisation, intervention allocation, outcome measurement or statistical analysis.

**Supplementary Table B1. Summary of independent consultative advisor input**

| **Element** | **Description** |
| --- | --- |
| Advisors | Three independent advisors with previous surgical experience and relevant communication, counselling or public-facing professional backgrounds. |
| Timing | Three advisory meetings were held during the study period: beginning, midpoint and end of the project. |
| Scope of input | Clarity, acceptability, patient relevance and patient-facing quality control of the communication protocol and associated patient-facing materials. |
| Role boundaries | Consultative input only; advisors did not alter trial conduct, randomisation, outcome measurement or statistical analysis. |
| Recommendation | The advisors recommended broader public review of the final findings before manuscript finalisation. |

**Supplementary Table B2. Summary of advisory group procedures**

| **Element** | **Description** |
| --- | --- |
| Participants | Six individuals with previous elective surgery experience. |
| Setting | Face-to-face meeting in a neutral community setting. |
| Session duration | Approximately 90 minutes. |
| Session components | Plain-language presentation of study findings, completion of an 8-item structured feedback form and open discussion. |
| Consent and anonymity | Written informed consent was obtained. Participants were assigned anonymised codes; no names or directly identifying information are reported. |
| Reimbursement | Participants received modest reimbursement for time and travel expenses. |
| Quantitative analysis | Likert-type ratings were summarised descriptively. |
| Qualitative analysis | Open-ended responses were transcribed, independently coded by two researchers and resolved by consensus. Themes were derived inductively using Braun and Clarke’s six-phase thematic analysis framework. |

**Supplementary Box B1. Patient and Public Advisory Group informed consent form**

| **Item** | **Text used in the consent form** |
| --- | --- |
| Study title | Structured AI Communication About Artificial Intelligence in Perioperative Care - Public Feedback Meeting |
| Date of meeting | 26 April 2026 |
| Venue | Neutral community meeting room [location anonymised] |
| Why are you invited? | The research team wishes to learn from individuals who have previously undergone elective surgery. We will share our preliminary study findings and ask for honest feedback on whether the results and recommendations are clear, useful and relevant for patients. |
| What will happen during the meeting? | Brief presentation of study results; completion of an 8-item feedback form; open discussion. Total duration: approximately 90 minutes. |
| Confidentiality and anonymity | Responses will be treated anonymously. No names, addresses or identifying information will appear in any report or publication. A unique participant code will be used for all records. |
| Voluntary participation | Participation is entirely voluntary. Participants may withdraw at any time without giving a reason and without any negative consequences. |
| Reimbursement | Participants will receive modest reimbursement for time and travel expenses. |
| Data storage | All anonymous data will be stored securely and accessed only by the research team. |
| Participant statement | I have read and understood the above information. I have had the opportunity to ask questions. I agree to take part in this public advisory group meeting. Participant code: _______. Signature: _______. Date: _______. |

**Supplementary Table B3. Public advisory group participant demographics and previous surgery experience**

| **ID** | **Age** | **Sex** | **Education** | **Previous surgery (year)** | **Anaesthesia type** |
| --- | --- | --- | --- | --- | --- |
| P01 | 41 | Female | University | Thyroidectomy (2023) | General |
| P02 | 68 | Male | High school | Cholecystectomy (2022) | General |
| P03 | 53 | Female | Postgraduate | Hysterectomy (2024) | Spinal |
| P04 | 62 | Female | Primary school | Knee replacement (2021) | Spinal |
| P05 | 49 | Male | University | Umbilical hernia (2023) | General |
| P06 | 54 | Female | High school | Carpal tunnel release (2024) | Local |

Note. Participant identifiers are anonymised. Directly identifying information was removed.

**Supplementary Table B4. Quantitative feedback form and descriptive summary**

| **Item** | **Statement** | **Your rating** | **For office use: mean (SD), n = 6** |
| --- | --- | --- | --- |
| Q1 | I found the study results (anxiety reduction) easy to understand. |  | 4.83 (0.41) |
| Q2 | A reduction in preoperative anxiety is clinically meaningful for patients. |  | 4.50 (0.55) |
| Q3 | The Practitioner Points are clear and actionable. |  | 4.67 (0.52) |
| Q4 | The Practitioner Points should be shortened (reverse-coded item). |  | 1.83 (0.75) |
| Q5 | The emphasis on uncertainty may increase patient anxiety. |  | 4.33 (0.82) |
| Q6 | The emphasis on calibrated trust is more helpful. |  | 4.67 (0.52) |
| Q7 | I support implementation of this communication protocol for patients. |  | 5.00 (0.00) |
| Q8 | Patient and public involvement should continue in future studies. |  | 4.83 (0.41) |

**Supplementary Box B2. Open-ended feedback prompts**

| **No.** | **Prompt** |
| --- | --- |
| 1 | How would you explain these findings to another patient waiting for surgery? |
| 2 | Would you change any of the Practitioner Points? If yes, how? |
| 3 | Do you have any other comments or suggestions? |

**Supplementary Table B5. Descriptive output for advisory group feedback**

| **Variable** | **N** | **Mean** | **Std. Deviation** | **Minimum** | **Maximum** |
| --- | --- | --- | --- | --- | --- |
| Q1 | 6 | 4.83 | 0.408 | 4 | 5 |
| Q2 | 6 | 4.50 | 0.548 | 4 | 5 |
| Q3 | 6 | 4.67 | 0.516 | 4 | 5 |
| Q4_rev | 6 | 1.83 | 0.753 | 1 | 3 |
| Q5 | 6 | 4.33 | 0.816 | 3 | 5 |
| Q6 | 6 | 4.67 | 0.516 | 4 | 5 |
| Q7 | 6 | 5.00 | 0.000 | 5 | 5 |
| Q8 | 6 | 4.83 | 0.408 | 4 | 5 |

Note. Valid N (listwise) = 6. Q4_rev refers to the reverse-coded item. All ratings used a 1-5 Likert-type response scale.

**Supplementary Table B6. Themes derived from open-ended public feedback**

| **Theme** | **Frequency** | **Illustrative evidence / analytic note** |
| --- | --- | --- |
| Small numerical reductions are perceived as clinically meaningful | 5/6 | Participants stated that even a modest reduction in anxiety would be noticeable and valuable before surgery. |
| Practitioner Points need to be shorter and more directive | 4/6 | Several participants found the original Practitioner Points too wordy for a clinical setting and preferred imperative wording. |
| The word uncertainty is anxiety-provoking | 5/6 | A strong preference emerged for emphasising calibrated trust, professional accountability and human oversight. |
| Spontaneous concern about data privacy | 2/6 | Two participants raised concerns about how patient data are used by AI systems; this was documented for future research because data governance was outside the scope of the communication trial. |

**Supplementary Table B7. Manuscript refinements informed by advisory group feedback**

| **Manuscript component** | **Change made / rationale** |
| --- | --- |
| Discussion section | The emphasis was framed as calibrated trust and interpretive preparedness rather than simple uncertainty reduction. |
| Practitioner Points | The points were shortened and rewritten in imperative style. |
| Data privacy suggestion | Not incorporated as a trial claim because data governance was outside the defined scope; documented for transparency and future research. |
| Other changes | No changes were made to trial design, outcome definition, allocation or statistical analysis. |
| Participant quotation guiding final revision | P06: “Trust is better. I trust my doctor, not the AI.” |

**Supplementary Table B8. GRIPP2 short-form reporting of public contribution**

| **GRIPP2 item** | **Application in this study** |
| --- | --- |
| Aim | To improve clarity, acceptability, patient-facing relevance and interpretation of the communication protocol and findings. |
| Methods | Independent consultative advisors contributed during the study; a public advisory group reviewed findings after analysis. |
| Results | Feedback supported calibrated trust, shorter practitioner-facing wording and explicit professional accountability. |
| Discussion | The manuscript acknowledges that involvement was consultative rather than full co-design. |
| Reflections | Future studies should use larger and more diverse patient/public advisory panels, cognitive interviews and co-design workshops. |

# **Section C. Outcome Measures, Item-Level Scoring and Data Quality Procedures**

All multi-item scale scores used in inferential analyses were reconstructed from item-level responses before analysis. This approach ensured consistent scoring direction, possible ranges and reproducibility across participants and time points. In response to peer review, this section now also reports the full item wording and response anchors of the three study-specific proximal indicators (Table C5) and an explicit statement of their construct limitations (Table C6).

**Supplementary Table C1. Outcome measures and scoring rules**

| **Measure** | **Items** | **Range** | **Direction** | **Timing** | **Analysis use** |
| --- | --- | --- | --- | --- | --- |
| APAIS anxiety | 4 | 4-20 | Higher = greater anxiety | T0, T1, T2 | Recomputed from item-level responses |
| Perceived control | 8 | 8-40 | Higher = greater perceived control | T0, T1, T2 | Recomputed from item-level responses |
| eHEALS | 8 | 8-40 | Higher = greater eHealth literacy | T0 | Recomputed from item-level responses |
| AI-related uncertainty | 4 | 4-20 | Higher = greater uncertainty | T1 | Study-specific proximal indicator |
| Information clarity | 4 | 4-20 | Higher = greater clarity | T1 | Study-specific proximal indicator |
| Trust in clinical team | 4 | 4-20 | Higher = greater trust | T1 | Study-specific proximal indicator |
| AI familiarity | 1 | 1-5 | Higher = greater perceived familiarity | T0 | Single-item baseline variable |
| Internet health information seeking | 1 | Low/Moderate/High | Higher category = more frequent seeking | T0 | Categorical baseline descriptor |

**Supplementary Table C2. Data scoring audit**

| **Scale** | **Stored total used?** | **Recomputed score used?** | **Reason** |
| --- | --- | --- | --- |
| APAIS anxiety | No | Yes | Ensured consistent scoring direction and possible range across T0, T1 and T2. |
| Perceived control | No | Yes | Ensured item-level reproducibility and consistent construction of the 8-item score. |
| eHEALS | No | Yes | Ensured item-level reproducibility and consistent construction of the 8-item score. |
| AI-related uncertainty | No | Yes | Ensured consistent construction of the 4-item proximal indicator. |
| Information clarity | No | Yes | Ensured consistent construction of the 4-item proximal indicator. |
| Trust in clinical team | No | Yes | Ensured consistent construction of the 4-item proximal indicator. |

**Supplementary Table C3. Study-specific proximal indicator domains**

| **Indicator** | **Item domains** |
| --- | --- |
| AI-related uncertainty | Uncertainty about the role of AI; whether AI replaces or supports clinical judgement; whether professional oversight remains in place; whether general AI-related information applies to the patient’s own care. |
| Information clarity | Clarity about AI’s supportive role; clarity that AI does not replace clinicians; clarity about professional oversight; clarity about the personal relevance of general AI-related information. |
| Trust in clinical team | Confidence that clinical decisions remain guided by health professionals; confidence that care is individualized; confidence that professional oversight is maintained; confidence in clinician-led interpretation. |

**Supplementary Table C4. Recomputed score quality-control summary**

| **Quality-control step** | **Application** |
| --- | --- |
| Range verification | All recomputed scale scores were checked against their theoretical minimum and maximum values. |
| Directionality verification | Higher-score interpretation was confirmed for each analysis scale before modelling. |
| Source of analysis scores | Inferential analyses used item-level reconstructed scores rather than pre-existing total-score fields. |
| Primary endpoint completeness | No T2 APAIS anxiety data were missing. |
| Reproducibility check | The score construction rules in Table C1 define the analysis variables used in all primary, secondary and exploratory analyses. |

**Supplementary Table C5. Item wording and response anchors for the three study-specific proximal indicators**

| **Indicator (scoring direction)** | **Item** | **Response anchors** |
| --- | --- | --- |
| AI-related uncertainty  (higher = greater uncertainty) | U1. I am unsure what role artificial intelligence plays in health care.  U2. I am unsure whether artificial intelligence replaces or supports the judgement of doctors and nurses.  U3. I am unsure whether health professionals still oversee decisions when artificial intelligence is involved.  U4. I am unsure whether general information about artificial intelligence applies to my own care. | 1 = strongly disagree  2 = disagree  3 = neither agree nor disagree  4 = agree  5 = strongly agree  Total = sum of 4 items (range 4–20) |
| Information clarity  (higher = greater clarity) | C1. It is clear to me that artificial intelligence may support, but does not replace, the clinical team.  C2. It is clear to me who is responsible for the decisions about my care.  C3. It is clear to me that health professionals oversee any information that is used in my care.  C4. It is clear to me how general information about artificial intelligence relates to my own situation. | 1 = strongly disagree  2 = disagree  3 = neither agree nor disagree  4 = agree  5 = strongly agree  Total = sum of 4 items (range 4–20) |
| Trust in the clinical team  (higher = greater trust) | T1. I am confident that decisions about my care remain guided by health professionals.  T2. I am confident that my care is planned for me as an individual.  T3. I am confident that professional oversight of my care is maintained.  T4. I am confident in my clinical team's interpretation of the information relevant to my care. | 1 = strongly disagree  2 = disagree  3 = neither agree nor disagree  4 = agree  5 = strongly agree  Total = sum of 4 items (range 4–20) |

Note. Items were administered in Turkish; the wording shown is the English-language manuscript version. Each indicator is the unweighted sum of its four items (range 4–20). No item is reverse-scored. Item-level descriptive statistics, internal consistency and distributional behaviour are reported in Supplementary Table E1.

**Supplementary Table C6. Construct limitations of the three study-specific proximal indicators**

| **Constraint** | **Description and consequence for interpretation** |
| --- | --- |
| Criterion contamination | Item content deliberately mirrors the five components of the intervention script. A participant who recalls what was said can score highly without any change in understanding, and the comparator arm was never exposed to this content. A large between-group difference is therefore close to guaranteed by design and cannot be read as a clinical effect. |
| No independent validation | The indicators were developed for this trial. No factor-analytic, test–retest or convergent-validity evidence is available, and they have not been evaluated in any other sample. |
| Modest internal consistency | Cronbach's α = .633 (AI-related uncertainty), .720 (information clarity) and .619 (trust), all below the conventional .80 threshold for individual-level inference (Table E1). |
| Distributional behaviour | Mild ceiling responding for information clarity (4.2%) and trust (3.3%); no floor effects. Skewness and kurtosis were within conventional bounds (Table E1). |
| Construct limitation: absence of doubt ≠ accuracy of belief | The AI-related uncertainty items index how uncertain a participant *feels*, not whether their beliefs are correct. A participant who concluded that AI is definitive and already validated would also score low. Distinguishing calibrated understanding from misplaced certainty requires items with objectively correct answers, which were not collected (Section 4.4 of the main manuscript). |
| Status in the analysis | Reported throughout as proximal communication-uptake indicators — evidence that the intended message was received — and never as independent patient-reported outcome measures or clinical endpoints. |

Note. These study-specific measures are interpreted as proximal communication-uptake indicators rather than validated independent clinical endpoints.

# **Section D. Extended Baseline, Clinical and Process Characteristics**

**Supplementary Table D1. Screening and non-enrolment log**

| Stage | n | Clarification |
| --- | --- | --- |
| Assessed for eligibility | 138 | Complete consecutive series of adult elective surgery patients approached between 05 February and 25 April 2026; no pre-screening filter was applied. |
| Excluded before randomization | 18 |  |
| Did not meet eligibility criteria | 11 | Recorded reasons comprised inability to complete study questionnaires; cognitive or communication limitations precluding informed participation; urgent or time-critical circumstances preventing a brief communication session; age below 18 years; or other clinical limitations. Reason-specific counts were not retained in the de-identified analysis dataset. |
| Declined to participate | 7 | Reasons for declining were not recorded. |
| Randomized | 120 | Allocated 1:1 to structured AI communication (n = 60) or attention-control supportive communication (n = 60). |

Note. The 138 patients assessed for eligibility represent the complete consecutive series approached during the recruitment window. The de-identified analysis dataset retained the total number of ineligible patients and the recorded categories of ineligibility, but not category-specific counts; no counts were imputed.

**Supplementary Table D2. Baseline characteristics using recomputed analysis scores**

| **Variable** | **Structured AI communication (n = 60)** | **Attention-control supportive communication (n = 60)** | **SMD** |
| --- | --- | --- | --- |
| Age, years | 44.73 (12.60) | 46.62 (14.35) | -0.14 |
| Sex |  |  | 0.10 |
| Female | 35 (58.3) | 32 (53.3) |  |
| Male | 25 (41.7) | 28 (46.7) |  |
| Previous surgery |  |  | 0.11 |
| Yes | 17 (28.3) | 14 (23.3) |  |
| No | 43 (71.7) | 46 (76.7) |  |
| Chronic disease |  |  | 0.05 |
| Yes | 9 (15.0) | 8 (13.3) |  |
| No | 51 (85.0) | 52 (86.7) |  |
| Education level |  |  | 0.24 |
| Primary | 16 (26.7) | 22 (36.7) |  |
| Secondary | 18 (30.0) | 19 (31.7) |  |
| University | 26 (43.3) | 19 (31.7) |  |
| Surgery type |  |  | 0.32 |
| General surgery | 24 (40.0) | 19 (31.7) |  |
| Urology | 15 (25.0) | 19 (31.7) |  |
| Orthopedics | 19 (31.7) | 15 (25.0) |  |
| Gynecology | 2 (3.3) | 7 (11.7) |  |
| Anaesthesia type |  |  | 0.30 |
| General | 35 (58.3) | 26 (43.3) |  |
| Regional | 21 (35.0) | 26 (43.3) |  |
| Local/Sedation | 4 (6.7) | 8 (13.3) |  |
| Expectancy score (1-5) | 3.49 (0.73) | 3.20 (0.70) | 0.40 |
| AI familiarity (1-5) | 2.45 (0.89) | 2.45 (0.93) | 0.00 |
| eHEALS T0 | 27.32 (4.58) | 27.20 (4.87) | 0.02 |
| APAIS anxiety T0 | 11.62 (2.55) | 12.27 (2.88) | -0.24 |
| Perceived control T0 | 21.68 (3.84) | 22.20 (3.86) | -0.13 |
| VAS pain postop day 1 | 3.60 (1.61) | 3.88 (1.51) | -0.18 |

Note. Continuous variables are mean (SD); categorical variables are n (%). For multi-category variables, the displayed SMD is the maximum absolute category-specific standardised difference. Several baseline and process descriptors exceeded 0.10 and are therefore transparently documented for sensitivity analyses rather than described as perfectly balanced.

**Supplementary Table D3. Process and delivery checks**

| **Process variable** | **Structured AI communication** | **Attention-control supportive communication** | **Comment** |
| --- | --- | --- | --- |
| Major protocol deviations | 0 | 0 | None recorded. |
| Randomisation deviations | 0 | 0 | None recorded. |
| Allocation concealment adequate | 60/60 | 60/60 | All records indicated concealed allocation. |
| Missing primary endpoint | 0 | 0 | Complete primary endpoint follow-up. |
| Session duration, minutes | 6.01 (0.78) | 5.22 (0.66) | SMD = 1.09; duration was recorded and adjusted in process sensitivity analysis. |
| T1-T2 interval, minutes | 19.57 (4.02) | 17.61 (4.13) | SMD = 0.48; interval was recorded and adjusted in process sensitivity analysis. |

**Supplementary Table D4. Provider distribution by study group**

| **Provider code** | **Structured AI communication** | **Attention-control supportive communication** |
| --- | --- | --- |
| N1 | 10 | 10 |
| N2 | 10 | 10 |
| N3 | 10 | 10 |
| N4 | 10 | 10 |
| N5 | 10 | 10 |
| N6 | 10 | 10 |

Note. Provider code was included in the process-adjusted sensitivity analysis as a fixed categorical term.

# **Section E. Psychometric and Distributional Analyses**

**Supplementary Table E1. Internal consistency and distributional properties**

| **Scale** | **Cronbach alpha** | **Floor %** | **Ceiling %** | **Skewness** | **Kurtosis** |
| --- | --- | --- | --- | --- | --- |
| APAIS T0 | 0.542 | 0.0 | 0.0 | 0.114 | -0.745 |
| APAIS T1 | 0.760 | 0.0 | 0.0 | 0.284 | -0.624 |
| APAIS T2 | 0.718 | 0.0 | 0.0 | 0.258 | -0.422 |
| Perceived Control T0 | 0.513 | 0.0 | 0.0 | -0.176 | -0.731 |
| Perceived Control T1 | 0.661 | 0.0 | 0.0 | -0.146 | -0.406 |
| Perceived Control T2 | 0.596 | 0.0 | 0.0 | -0.127 | -0.487 |
| eHEALS T0 | 0.711 | 0.0 | 0.0 | 0.069 | -0.240 |
| AI uncertainty T1 | 0.633 | 3.3 | 0.0 | 0.065 | -0.842 |
| Information clarity T1 | 0.720 | 0.0 | 4.2 | -0.244 | -0.913 |
| Trust clinical team T1 | 0.619 | 0.0 | 3.3 | -0.210 | -0.244 |

Note. Cronbach alpha values describe internal consistency in this dataset. Internal consistency was below the conventional threshold for the APAIS anxiety subscale at baseline (.542) and for the perceived control scale at every assessment point (.513 to .661), and was modest for all three study-specific proximal indicators (.619 to .720). These endpoints are therefore interpreted supportively in the main manuscript, alongside baseline-adjusted modelling, HC3 robust intervals and bootstrap sensitivity estimates. The study-specific proximal indicators are reported as communication-uptake indicators rather than as fully validated standalone patient-reported outcome measures; their construct limitations are set out in Table C6.

**Supplementary Table E2. Interpretation of psychometric checks**

| **Finding** | **Interpretation** |
| --- | --- |
| No floor or ceiling effect for the primary endpoint | Supports use of APAIS anxiety for detecting pre-surgery differences. |
| Moderate-to-good internal consistency for established scales | Supports item-level reconstruction of APAIS, perceived control and eHEALS in the analysis dataset. |
| Proximal indicators showed strong group separation | Consistent with communication uptake; interpreted cautiously because indicator content is close to the intervention content. |
| Residual normality tests were statistically significant in some models | With n = 120, inference was supported by model diagnostics, HC3 robust intervals and bootstrap sensitivity estimates. |

# **Section F. Extended Primary, Secondary and Proximal Outcome Analyses**

**Supplementary Table F1. Primary and key secondary outcome analyses**

| **Analysis** | **Estimate/MD** | **95% CI lower** | **95% CI upper** | **p** | **Model/Note** |
| --- | --- | --- | --- | --- | --- |
| Primary: APAIS T2 ANCOVA | -2.56 | -2.87 | -2.26 | < .001 | Treatment + baseline APAIS |
| Primary HC3 robust | -2.56 | -2.88 | -2.25 | < .001 | HC3 robust SE |
| Primary bootstrap CI | -2.56 | -2.86 | -2.26 |  | Participant-level bootstrap CI |
| APAIS T2 unadjusted | -3.20 |  |  | < .001 | Welch mean difference |
| APAIS change score | 2.55 |  |  | < .001 | T0-T2 reduction difference; positive favours intervention |
| Key secondary: perceived control T2 ANCOVA | 3.73 | 3.34 | 4.12 | < .001 | Treatment + baseline perceived control |
| Perceived control T2 unadjusted | 3.22 |  |  | < .001 | Welch mean difference |
| Perceived control change score | 3.73 |  |  | < .001 | T0-T2 change difference |

Note. Negative values for APAIS favour structured AI communication; positive values for perceived control favour structured AI communication.

**Supplementary Table F2. Proximal communication-response indicators**

| **Outcome** | **Structured AI communication** | **Attention-control supportive communication** | **Unadjusted MD (95% CI)** | **p** | **Cohen's d** | **Mann-Whitney sensitivity** |
| --- | --- | --- | --- | --- | --- | --- |
| AI-related uncertainty T1 | 7.33 (1.64) | 11.70 (1.83) | -4.37 (-4.99 to -3.74) | < .001 | -2.51 | U = 127.5; p < .001 |
| Information clarity T1 | 17.20 (1.65) | 12.80 (2.15) | 4.40 (3.71 to 5.09) | < .001 | 2.30 | U = 3383.0; p < .001 |
| Trust in clinical team T1 | 16.20 (1.71) | 12.43 (2.13) | 3.77 (3.07 to 4.47) | < .001 | 1.95 | U = 3329.0; p < .001 |

Note. AI-related uncertainty is scored higher = greater uncertainty. Structured AI communication was associated with substantially lower AI-related uncertainty and higher information clarity/trust.

**Supplementary Table F3. Primary estimand specification**

| **Component** | **Specification** |
| --- | --- |
| Population | All randomized participants analysed according to allocated group. |
| Treatment condition | Structured AI communication. |
| Comparator | Time-matched attention-control supportive communication. |
| Outcome | T2 APAIS anxiety recomputed from item-level responses. |
| Summary measure | Baseline-adjusted mean difference. |
| Primary model | ANCOVA with treatment group and baseline APAIS anxiety. |
| Missing data handling | No primary endpoint data were missing; no imputation was required. |

# **Section G. Clinically Meaningful Response and Threshold Analyses**

**Supplementary Table G1. Clinically meaningful anxiety response**

| **Responder outcome** | **Structured AI communication** | **Attention-control supportive communication** | **Risk difference (95% CI)** | **NNT (95% CI)** | **Fisher p** |
| --- | --- | --- | --- | --- | --- |
| APAIS reduction ≥ 2 points | 48/60 (80.0) | 1/60 (1.7) | 78.3% (64.5 to 86.6) | 1.28 (1.15 to 1.55) | < .001 |
| APAIS reduction ≥ 1 point | 60/60 (100.0) | 8/60 (13.3) | 86.7% (74.3 to 93.1) | 1.15 (1.07 to 1.35) | < .001 |
| Moved below APAIS ≥11 threshold | 21/40 (52.5) | 2/44 (4.5) | 48.0% (29.6 to 62.9) | 2.09 (1.59 to 3.38) | < .001 |

Note. APAIS reduction = T0 minus T2. Risk difference used Newcombe confidence intervals. NNT confidence limits were derived from the risk-difference interval; interpret NNT conservatively for exploratory thresholds.

**Supplementary Table G2. Responder-analysis interpretation rules**

| **Issue** | **Analytic decision** |
| --- | --- |
| Responder definition | At least 2-point reduction in APAIS anxiety from baseline to T2 was the primary clinically meaningful threshold. |
| Secondary threshold | At least 1-point reduction was analysed as a more liberal supporting threshold. |
| Clinical-threshold analysis | Movement below the APAIS anxiety threshold was restricted to participants with baseline APAIS anxiety >= 11. |
| Sparse control events | Risk difference and NNT were preferred; risk ratio was avoided as the primary effect estimate when event counts were very small. |
| Inference | Fisher exact p values were used for responder comparisons. |

# **Section H. Sensitivity Analyses and Model Diagnostics**

**Supplementary Table H1. Sensitivity analyses for the primary outcome**

| **Model** | **Adjusted MD** | **95% CI** | **p / diagnostic** | **AIC** |
| --- | --- | --- | --- | --- |
| Baseline-adjusted ANCOVA | -2.56 | -2.87 to -2.26 | < .001 | 299.5 |
| ANCOVA + prognostic covariates | -2.55 | -2.85 to -2.25 | < .001 | 296.7 |
| Process-adjusted model | -2.71 | -3.06 to -2.36 | < .001 | 303.4 |
| HC3 robust SE | -2.56 | -2.88 to -2.25 | < .001 |  |
| Bootstrap | -2.56 | -2.86 to -2.26 | Bias 0.003 |  |

Note. Process-adjusted model included session duration, T1-T2 interval and provider code. Prognostic covariates were age, sex, AI familiarity, eHEALS and expectancy score.

**Supplementary Table H2. Model diagnostics**

| **Diagnostic** | **p value** | **Interpretation** |
| --- | --- | --- |
| Primary residual Shapiro-Wilk | .014 | Statistically significant; robust and bootstrap sensitivity estimates retained the same conclusion. |
| Primary Levene test | .066 | No strong evidence of residual variance heterogeneity at alpha .05. |
| Group x baseline anxiety interaction | .184 | F = 1.78; homogeneity of regression slopes was not rejected. |
| Perceived-control residual Shapiro-Wilk | .004 | Statistically significant; interpretation supported by large baseline-adjusted effect and robust sensitivity. |
| Perceived-control Levene test | .022 | Some heterogeneity; secondary endpoint interpreted supportively. |
| Group x baseline perceived control interaction | .763 | F = 0.09. |

**Supplementary Table H3. Covariates included in sensitivity models**

| **Model** | **Covariates** |
| --- | --- |
| Primary model | Treatment group and baseline APAIS anxiety. |
| Prognostic-covariate model | Treatment group, baseline APAIS anxiety, age, sex, AI familiarity, eHEALS and expectancy score. |
| Process-adjusted model | Treatment group, baseline APAIS anxiety, session duration, T1-T2 interval and provider code. |
| Robust model | Primary model with HC3 heteroscedasticity-consistent standard errors. |
| Bootstrap model | Primary model resampled at participant level. |

**Supplementary Table H4. Post hoc bias-threshold and responder-misclassification sensitivity analyses**

Panel A. Bias-threshold (tipping-point) analysis for the primary continuous endpoint

| **Scenario** | **Required systematic bias (APAIS points)** | **In baseline SD units (SD = 2.72)** | **Resulting conclusion** |
| --- | --- | --- | --- |
| Adjusted MD falls below the 2-point minimum clinically important difference | 0.56 | 0.21 | Difference remains statistically evident but is no longer clinically meaningful. |
| Upper limit of the 95% CI reaches the null | 2.26 | 0.83 | Difference is no longer statistically distinguishable from zero. |
| Adjusted MD reduced to zero | 2.56 | 0.94 | Difference is entirely explained by bias. |

Panel B. Responder misclassification at the ≥2-point APAIS threshold

| **Scenario** | **Structured AI communication** | **Attention-control supportive communication** | **Risk difference, % (95% CI)** |
| --- | --- | --- | --- |
| Observed | 48/60 (80.0%) | 1/60 (1.7%) | 78.3 (64.5 to 86.6) |
| S1: 10 intervention responders reclassified as non-responders; comparator responders tripled | 38/60 (63.3%) | 3/60 (5.0%) | 58.3 (43.0 to 69.9) |
| S2: 25% of intervention responders false positive; comparator responders × 6 | 36/60 (60.0%) | 6/60 (10.0%) | 50.0 (33.8 to 62.6) |
| S3: intervention responder rate reduced to 50%; comparator responders × 6 | 30/60 (50.0%) | 6/60 (10.0%) | 40.0 (24.1 to 53.4) |
| S4: intervention responder rate halved; comparator responders × 6 | 24/60 (40.0%) | 6/60 (10.0%) | 30.0 (14.7 to 43.7) |
| S5: intervention responder rate halved; comparator responders × 12 (most extreme scenario examined) | 24/60 (40.0%) | 12/60 (20.0%) | 20.0 (3.6 to 35.0) |

Panel C. E-values for the responder risk ratio

| **Quantity** | **Value** | **Interpretation** |
| --- | --- | --- |
| Responder risk ratio (≥2-point threshold) | 48.0 (95% CI, 6.84 to 336.6) | Computed from 48/60 versus 1/60 using the log-risk-ratio method. |
| E-value for the point estimate | 95.5 | An unmeasured source of bias would need to be associated with both allocation and responder status by a risk ratio of at least 95.5 to explain away the observed association. |
| E-value for the lower confidence limit | 13.2 | The corresponding threshold for shifting the confidence interval to include the null. |

Note. Both analyses were post hoc and are identified as such in the main manuscript. Panel A expresses how large a systematic non-specific response bias favouring the intervention arm (for example, from expectancy, social desirability or measurement reactivity) would need to be, in APAIS points, to change each stated conclusion; the baseline standard deviation used is the pooled T0 value (2.72). Panel B recomputes the risk difference under progressively extreme misclassification assumptions using Newcombe confidence intervals, the same method used for the primary responder analysis. Panel C reports E-values (VanderWeele & Ding, 2017); E-values were developed for unmeasured confounding rather than measurement bias and are given here only as an order-of-magnitude benchmark. None of these analyses can exclude bias sufficient to inflate the size of the observed effect; they address only whether bias could plausibly reverse its direction.

**Supplementary Table H5. Provider (nurse) clustering check**

| **Model** | **Fixed effect of allocation on APAIS T2 (95% CI)** | **Provider ICC** | **Note** |
| --- | --- | --- | --- |
| Primary ANCOVA (no provider term) | −2.56 (−2.87 to −2.26) | — | Reproduced from Table H1. |
| ANCOVA + provider as fixed categorical term (process-adjusted model) | −2.71 (−3.06 to −2.36) | — | Reproduced from Table H1. |
| Linear mixed model with provider random intercept | «__ (__ to __)» | «__» | Clustering check requested at peer review. Fitted on the analysis dataset with the same covariate as the primary model. |

Note. Six nurses delivered the sessions and each delivered both conditions to 10 participants per arm (Table D3), so provider is orthogonal to allocation by design and cannot confound the treatment comparison; residual clustering could nevertheless make the reported confidence intervals slightly too narrow. Model specification: lmer(APAIS_T2 ~ group + APAIS_T0 + (1 | provider)) in R, or MIXED APAIS_T2 BY group WITH APAIS_T0 /RANDOM = INTERCEPT | SUBJECT(provider) in SPSS. Cells marked «__» are to be completed from the analysis dataset before submission.

# **Section I. Mixed-Effects Anxiety Trajectory Analysis**

**Supplementary Table I1. Mixed-effects model fixed effects for APAIS trajectory**

| **Parameter** | **Estimate** | **95% CI lower** | **95% CI upper** | **p** |
| --- | --- | --- | --- | --- |
| Intercept | 12.27 | 11.58 | 12.96 | < .001 |
| C(Time)[T.T1] | 0.10 | -0.14 | 0.34 | .414 |
| C(Time)[T.T2] | 0.22 | -0.02 | 0.46 | .077 |
| G | -0.65 | -1.63 | 0.33 | .192 |
| G:C(Time)[T.T1] | -3.30 | -3.64 | -2.96 | < .001 |
| G:C(Time)[T.T2] | -2.55 | -2.89 | -2.21 | < .001 |

Note. Model included fixed effects for group, time and group-by-time interaction with a participant-level random intercept. ML AIC = 1213.4.

**Supplementary Table I2. Anxiety means and simple trajectory contrasts**

| **Time/contrast** | **Structured AI communication** | **Attention-control supportive communication** | **Difference/change** | **p** |
| --- | --- | --- | --- | --- |
| T0 | 11.62 (2.55) | 12.27 (2.88) | -0.65 | .193 |
| T1 | 8.42 (2.46) | 12.37 (3.00) | -3.95 | < .001 |
| T2 | 9.28 (2.55) | 12.48 (3.01) | -3.20 | < .001 |
| --- | --- | --- | --- | --- |
| T0 to T1 | Structured AI communication |  | -3.20 (-3.43 to -2.97) | < .001 |
| T1 to T2 | Structured AI communication |  | 0.87 (0.54 to 1.20) | < .001 |
| T0 to T2 | Structured AI communication |  | -2.33 (-2.57 to -2.10) | < .001 |
| T0 to T1 | Attention-control supportive communication |  | 0.10 (-0.10 to 0.30) | .321 |
| T1 to T2 | Attention-control supportive communication |  | 0.12 (-0.15 to 0.38) | .382 |
| T0 to T2 | Attention-control supportive communication |  | 0.22 (0.03 to 0.41) | .027 |

Note. Top rows show between-group means by time; lower rows show within-group changes from paired analyses.

# **Section J. Exploratory Moderation Analyses**

**Supplementary Table J1. Moderation of the intervention effect on T2 APAIS anxiety**

| **Moderator** | **Interaction beta** | **Interaction 95% CI** | **p-interaction** | **Moderator level** | **Marginal MD (95% CI)** |
| --- | --- | --- | --- | --- | --- |
| eHealth literacy | 0.084 | 0.022 to 0.146 | .008 | Low (-1 SD) | -2.97 (-3.38 to -2.55) |
|  |  |  |  | Mean | -2.57 (-2.86 to -2.28) |
|  |  |  |  | High (+1 SD) | -2.18 (-2.59 to -1.77) |
| AI familiarity | 0.107 | -0.223 to 0.438 | .521 | Low (-1 SD) | -2.67 (-3.09 to -2.24) |
|  |  |  |  | Mean | -2.57 (-2.87 to -2.27) |
|  |  |  |  | High (+1 SD) | -2.47 (-2.90 to -2.05) |
| Baseline anxiety | -0.076 | -0.188 to 0.037 | .184 | Low (-1 SD) | -2.36 (-2.79 to -1.93) |
|  |  |  |  | Mean | -2.57 (-2.87 to -2.26) |
|  |  |  |  | High (+1 SD) | -2.77 (-3.21 to -2.34) |

Note. Continuous moderators were mean-centred. Negative marginal MD values favour structured AI communication. These analyses are exploratory and not powered for definitive subgroup inference.

**Supplementary Table J2. Safeguards for exploratory moderation interpretation**

| **Safeguard** | **Application** |
| --- | --- |
| Prespecified exploratory status | Moderation analyses were interpreted as hypothesis-generating. |
| Continuous moderators | Moderators were analysed continuously and mean-centred before interaction testing. |
| Marginal effects | Effects were summarised at -1 SD, mean and +1 SD to avoid arbitrary dichotomisation. |
| Multiplicity | No multiplicity adjustment was applied; emphasis was placed on effect direction, interval width and plausibility. |

# **Section K. Exploratory Pathway-Consistency and Mediation Analyses**

**Supplementary Table K1. Exploratory pathway-consistency analysis**

| **Pathway candidate** | **Observed pattern** | **Interpretation** |
| --- | --- | --- |
| Information clarity | Structured AI communication improved information clarity at T1 and lower T2 anxiety remained evident. | Pattern supports the interpretation that clarity is a plausible proximal communication-response mechanism. |
| Trust in clinical team | Structured AI communication improved trust at T1 and lower T2 anxiety remained evident. | Pattern supports calibrated clinician-led trust as a plausible proximal pathway. |
| AI-related uncertainty | Structured AI communication reduced AI-related uncertainty in the analysis dataset. | This is consistent with the intervention’s interpretive-preparedness logic and should be framed as a communication response rather than independent construct validation. |
| Perceived control | Perceived control improved after baseline adjustment. | Consistent with the view that interpretive orientation and professional accountability can improve patient-centred preparedness. |

**Supplementary Table K2. Exploratory simultaneous mediation estimates**

| **Effect component** | **Estimate** | **Bootstrap 95% CI** | **Comment** |
| --- | --- | --- | --- |
| Information clarity pathway | 0.17 | -0.13 to 0.50 | Participant-level bootstrap interval. |
| Trust pathway | 0.06 | -0.24 to 0.41 | Participant-level bootstrap interval. |
| Direct effect after mediators | -2.80 | -3.29 to -2.34 | Participant-level bootstrap interval. |
| Total baseline-adjusted effect | -2.56 | -2.86 to -2.25 | Participant-level bootstrap interval. |

Note. Mediation was exploratory and should not be interpreted causally. Estimates are intended to support pathway consistency only.

# **Section L. Reporting Checklist Mapping and Reproducibility Materials**

**Supplementary Table L1. Reproducibility workflow**

| **Stage** | **Content** |
| --- | --- |
| Data import | The 120-participant analysis dataset was used as the analysis source. |
| Score reconstruction | APAIS, perceived control, eHEALS and proximal indicators were recomputed from item-level variables. |
| Primary model | ANCOVA estimated T2 APAIS anxiety by group with baseline APAIS anxiety adjustment. |
| Sensitivity checks | HC3 robust SE, covariate-adjusted model, process-adjusted model and bootstrap intervals. |

**Supplementary Table L2. Reporting checklist mapping**

| **Reporting item** | **Location in manuscript or supplement** |
| --- | --- |
| Trial design and allocation | Main Methods; Supplementary Sections A, F and H |
| Eligibility, setting and recruitment | Main Methods; participant flow in main manuscript |
| Intervention content | Supplementary Section A |
| Comparator content | Supplementary Section A |
| Patient/public contribution | Main Methods; Supplementary Section B |
| Outcome definitions and scoring | Main Methods; Supplementary Section C |
| Baseline and process characteristics | Main Table 1; Supplementary Section D |
| Primary and secondary analyses | Main Results; Supplementary Sections F-H |
| Responder and threshold analyses | Main Results; Supplementary Section G |
| Trajectory and exploratory analyses | Supplementary Sections I-K |
| Reproducibility and coding | Supplementary Section L |

**Supplementary Table L3. Variable coding and role in analysis**

| **Variable group** | **Examples** | **Coding/role** |
| --- | --- | --- |
| Treatment group | Treatment group | Intervention = 1; attention-control supportive communication = 0. |
| Primary outcome | APAIS_T2 | Continuous total score, 4-20; higher = greater anxiety. |
| Baseline covariate | APAIS_T0 | Continuous baseline anxiety adjustment for primary ANCOVA. |
| Key secondary outcome | PerceivedControl_T2 | Continuous total score, 8-40; higher = greater perceived control. |
| Digital-literacy variables | eHEALS_T0, AI_Familiarity_T0, Internet_Health_Info_Seeking | Baseline descriptors and exploratory moderators/covariates. |
| Process variables | Intervention_Duration_Min, Time_T1_T2_Min, Nurse_ID | Used in process-adjusted sensitivity analysis. |
| Clinical descriptors | Surgery_Type, Anesthesia_Type, VAS_Pain_Postop1 | Descriptive clinical context; not part of primary endpoint adjustment. |
| Responder variables | Responder_1pt, Responder_2pt | Derived from APAIS_T0 - APAIS_T2. |

**Supplementary Table L4. Reporting guidelines applied**

| **Guideline** | **Application in this trial** | **Location** |
| --- | --- | --- |
| CONSORT 2010 (Schulz et al., 2010) | Parallel-group randomized trial reporting. | Separate CONSORT checklist file; Figure 1. |
| CONSORT extension for non-pharmacological treatments (Boutron et al., 2017) | Description of the intervention and comparator, provider characteristics and training, delivery standardisation, and explicit reporting of what could and could not be blinded. | Main manuscript Sections 2.5–2.9; Supplementary Tables A1–A5, D2, D3. |
| TIDieR (Hoffmann et al., 2014) | Twelve-item description of both study conditions. | Supplementary Table A5. |
| GRIPP2 short form (Staniszewska et al., 2017) | Reporting of patient and public contribution. | Supplementary Table B8. |

Note. Reporting was mapped to the CONSORT extension for non-pharmacological treatments and the TIDieR checklist.
